# How Fiber Surface Topography Affects Interactions between Cells and Electrospun Scaffolds: A Systematic Review

**DOI:** 10.3390/polym14010209

**Published:** 2022-01-05

**Authors:** Alex Lopez Marquez, Iván Emilio Gareis, Fernando José Dias, Christoph Gerhard, María Florencia Lezcano

**Affiliations:** 1 Faculty of Engineering and Health, University of Applied Sciences and Arts, 37085 Göttingen, Germany; alexlopezmarquez@gmail.com (A.L.M.); christoph.gerhard@hawk.de (C.G.); 2 Laboratorio de Cibernética, Departamento de Bioingeniería, Facultad de Ingeniería, Universidad Nacional de Entre Ríos, Oro Verde 3100, Argentina; igareis@ingenieria.uner.edu.ar; 3 Research Centre for Dental Sciences CICO, Department of Integral Adults Dentistry, Dental School, Universidad de La Frontera, Temuco 4811230, Chile; fernando.dias@ufrontera.cl

**Keywords:** electrospun scaffold, topography, fiber surface, cell proliferation, cell adhesion, cell differentiation, nanoporosity, nanotopography, surface morphology

## Abstract

Electrospun scaffolds have a 3D fibrous structure that attempts to imitate the extracellular matrix in order to be able to host cells. It has been reported in the literature that controlling fiber surface topography produces varying results regarding cell–scaffold interactions. This review analyzes the relevant literature concerning in vitro studies to provide a better understanding of the effect that controlling fiber surface topography has on cell–scaffold interactions. A systematic approach following PRISMA, GRADE, PICO, and other standard methodological frameworks for systematic reviews was used. Different topographic interventions and their effects on cell–scaffold interactions were analyzed. Results indicate that nanopores and roughness on fiber surfaces seem to improve proliferation and adhesion of cells. The quality of the evidence is different for each studied cell–scaffold interaction, and for each studied morphological attribute. The evidence points to improvements in cell–scaffold interactions on most morphologically complex fiber surfaces. The discussion includes an in-depth evaluation of the indirectness of the evidence, as well as the potentially involved publication bias. Insights and suggestions about dose-dependency relationship, as well as the effect on particular cell and polymer types, are presented. It is concluded that topographical alterations to the fiber surface should be further studied, since results so far are promising.

## 1. Introduction

Electrospinning is a versatile technique to generate ultrathin fibers [1]. Electrospun nano- and microfibers have proven to be useful in several fields of application—e.g., filtration and protective material, electrical and optical applications, biomedical applications, sensors, and nanofiber reinforced composites [1,2]. One of the most important fields of application is the biomedical field [1,2]. Electrospun fibers are currently used in tissue engineering [3,4], drug release [3,5,6], wound dressing [7], and monitoring of the blood glucose level for the diagnosis of diabetes [8], among others.

Over the past few decades, this technology has been used to develop a vast variety of nanofibrous scaffolds for tissue engineering [4]. Electrospun scaffolds are known to be a prominently promising creation of tissue engineering. Since in tissue engineering, one implicit goal is to replicate living tissue, a substrate for cells must exist for them to effectively develop on. In nature, this role is fulfilled by the extracellular matrix (ECM). In order to imitate the ECM of humans, one approach is to emulate its hierarchical multiscale structures and complexity. This is where electrospun scaffolds shine, because they can achieve properties such as very thin fibers, large surface areas, and superior mechanical properties. Their ease of processing makes them an excellent candidate for large-scale production [2].

Although many scaffolds can substitute the ECM with different amounts of success, the gold standard for most tissue regeneration purposes is known to be autograft [9,10]. Nevertheless, autograft is not without its shortcomings: it is harvested from the patient, causing a permanent deficit in the donor site, the obtained tissue has varying quality and clinical outcomes have undesirable success rates [11]. This has caused tissue engineers to attempt to find alternatives, which have shown promising results so far [12,13].

Biomimetic hierarchical scaffolds can be created by electrospinning or 3D printing, which are the two main alternatives that currently compete in this field. Due to its more advanced development and wider publication [14], electrospinning was chosen as the focus for this review.

In the last few years, researchers have actively worked on identifying and improving the properties of electrospun scaffolds to optimize their capability to host cells. Favorable morphological characteristics can be difficult to identify because of the complexity of the structures and processes involved [15,16,17]. Mathematical and computational modeling have been shown to be very useful to sort this difficulty [15,16,17]. There are many approaches for functionalization of electrospun scaffolds, ranging from improving their mechanical properties to improving cell infiltration or biocompatibility [18], as well as the control of material, dimensions and chemical surface modification, among others [19]. One such improvement involves studying the effect of different surface structures of individual fibers on how well cells can develop on them. In order to do this, surface structures on fibers are elicited, and then different cell–scaffold interaction variables are measured, including adhesion, proliferation, and differentiation.

Changing the topography of electrospun scaffold fibers seems particularly attractive, since alternative methods to improve cell–scaffold interactions tend to be expensive and dedicated. Scaffolds with engineered fiber topography can be acquired by applying relatively small changes in manufacturing or post-processing. If these improve cell–scaffold interactions, then researchers will have a tool at their disposal that they can easily implement to functionalize their scaffolds. However, no review was found in the literature addressing this particular matter.

A review was found [20] which shortly touches upon feature elicitation, but it includes surface features in which other materials are involved. It also heavily focuses on core-shell electrospun nanofibers and other methodologies, without analyzing the cell–scaffold interactions. There are several feature elicitation methods discussed. One of the methods consists in controlling the formation of nanopores by adjusting the mixture of the polymer with a solvent or a non-solvent, causing what is known as nonsolvent induced phase separation (NSIPS). Further, temperature adjustments during the process-thermal induced phase separation (TIPS) and relative humidity changes, also known as vapor-induced phase separation (VIPS), are introduced. Another review [21] reported on the impact of micro-/nanotopography of surfaces and interfaces, in general terms, on differentiation of cells. In 2019, a review [22] described how electrospun scaffolds can be micropatterned, referring to the fact that they can be sectioned in to micrometric areas, which has an effect on drug release, cell patterning, and differentiation. The effect of topographically patterned surfaces on cell behavior was reviewed back in 2007 [23], and it was reported that osteoblasts saw improved differentiation with certain topographical cues. Finally, another review was found [24] that assessed the effects of electrospun scaffold patterning on cells, as well as computational models to predict this behavior. This patterning referred to any micro- or nanoscale patterning, including fiber alignment.

The aim of this review is to evaluate the current state of knowledge in the literature regarding the effect of any topographical modification of electrospun fiber surfaces on cell adhesion, proliferation and/or differentiation. Since researchers often use different cell lines, materials and fiber topography elicitation methods, this review will attempt to evaluate whether an effect exists independently of cell type, material, and topography elicitation method. Due to this, the format of a systematic review has been chosen, to reflect the current state of literature while minimizing risk of bias.

This systematic review will provide readers with an overview of the existing evidence; give an in-depth analysis of the applicability, generalizability, and quality of the evidence; and analyze potential biases involved in the process.

## 2. Materials and Methods

This systematic review follows the PRISMA statement [25] recommendations in order to reduce potential bias. PICO, a set of criteria that can improve search results [26], is used to establish search criteria and strategy. A protocol exists for this review and can be found online [27].

The eligibility criteria for the studies were based on PICO, and the following PICO criteria were selected:

- Problem: Fiber surface topography might affect cell development on electrospun scaffolds.
- Intervention: Modification of fiber surface topography.
- Comparison: Surface-modified scaffolds are compared to non-surface-modified scaffolds.
- Outcome: Changed cell adhesion, proliferation, or differentiation.

The databases searched were ScienceDirect, PubMed, Scopus, Web of Science, and Embase. For the search strategy, research only in the English language and published at any time was considered. The following string of keywords was used for the search, based on the PICO eligibility criteria:

The term “topography” is often used to describe whether the individual scaffold fibers are aligned [28]. Due to this, researchers interested in knowing the effect of fiber surface structures on cell–scaffold interactions may find that a search for “topography” yields a large number of results, few of which evaluate the intended question. This is the reason that “nanotopography”, and not “topography,” was used. The reason “differentiation” was not used is that a preliminary search showed that the highly numerous results were mostly unrelated to the objective of this review, and that many papers involving differentiation appeared even without the keyword, possibly due to the rest of the involved keywords.

The search was then performed, and any results found through the references were added to the list, after which duplicates were eliminated. The abstracts and titles of all articles were then screened and studies that followed the established PICO selection criteria were selected. Any other articles were excluded. The next step was to read the full-text articles and exclude, with justification, those that did not fulfil the selection criteria.

The risk of bias in individual studies (study-level bias) was evaluated using a modified version of the CASP qualitative study evaluation form [29]. Items not relevant to this systematic review were removed, and additional items and sources of bias were included.

The data from the selected articles was then collected. For this, all relevant data items were identified that could potentially be relevant to the proposed question. These items were added to a data extraction form, which was subsequently filled out for each article. The results were summarized in tabular form. The concise presentation of a tabular form, along with an explanation of the context, was deemed to be appropriate for the given data.

Risk of bias across studies (outcome-level bias as well as any other sources of bias) was evaluated using the methods described in the GRADE handbook [30], with an appraisal of the quality of evidence by outcome. AMSTAR, a tool to evaluate the methodological quality of systematic reviews [31], was used to evaluate potential bias sources in the systematic review process.

## 3. Results

### 3.1. Study Selection

**Figure 1:**
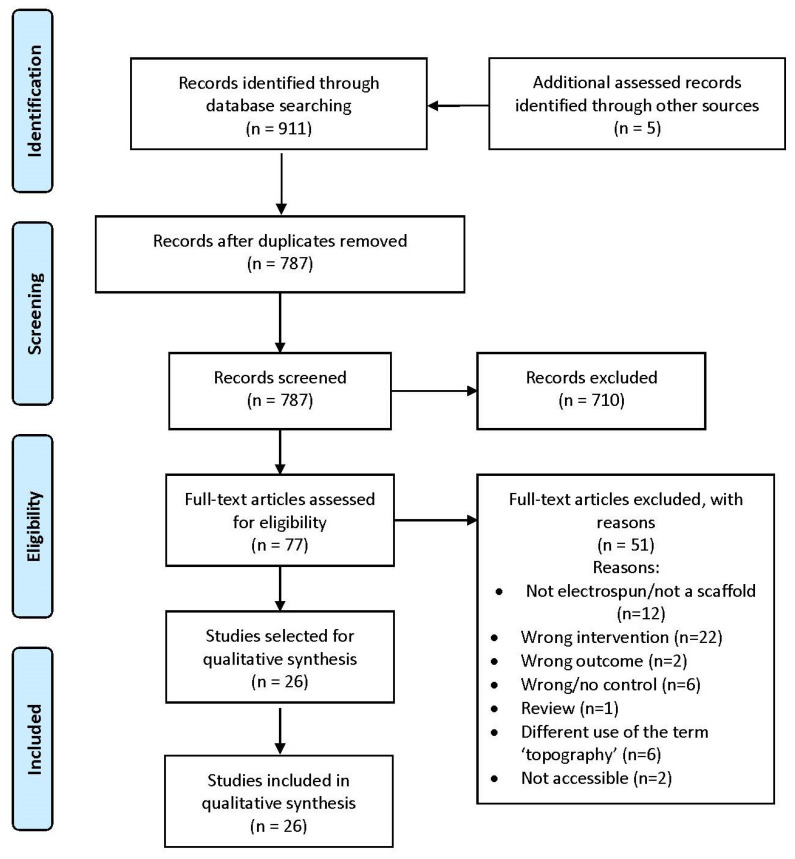
PRISMA flow diagram. Reused from [25] (CCBY-Licensed).

The search process is illustrated in Figure 1. It was performed on 7 January 2021, and delivered the following results:

- ScienceDirect (693 results, filtered by research articles);
- PubMed (36 results, filtered by English language);
- Scopus (58 results);
- Web of Science (98 results);
- Embase (26 results).

This amounts to a total of 911 results. Another five results were found in the references. By eliminating a total of 129 duplicates, 787 search results remained.

The abstracts and titles of all articles were then screened, and studies that followed the established PICO selection criteria were selected. The screening allowed for the exclusion of 710 articles, so that 77 full-text articles remained. The reason that approximately 90% of the screened articles were excluded is the strict interpretation of the PICO criteria, which is intended to prevent the inclusion of any article that is not strictly relevant to the question.

The next step was to read the full-text articles and exclude, with justification, those that did not fulfil the selection criteria. Fifty-one articles were excluded. The justification for the exclusions is listed in Supplementary Information File S1. Twenty-six full-text articles were selected [32,33,34,35,36,37,38,39,40,41,42,43,44,45,46,47,48,49,50,51,52,53,54,55,56,57]. It is worth noting that, at this point, it became apparent that cell viability was often measured in these studies, and it was added as an outcome.

### 3.2. Study Characteristics

The raw data extraction form can be seen in Supplementary Information File S2.

### 3.3. Risk of Bias within Studies

The results of the evaluation of risk of bias within studies can be seen in Table 1. Most of the studies have at least one source of risk of bias. Often, this is performance bias, which is induced by not controlling or purposely changing variables that can potentially affect the result. On the other hand, none of the studies presented any signs of detection bias (because the detection methods were not probable to affect the results).

### 3.4. Results of Individual Studies

The results of the data extraction can be seen in Supplementary Information File S3.

### 3.5. Synthesis of Results

**Figure 2:**
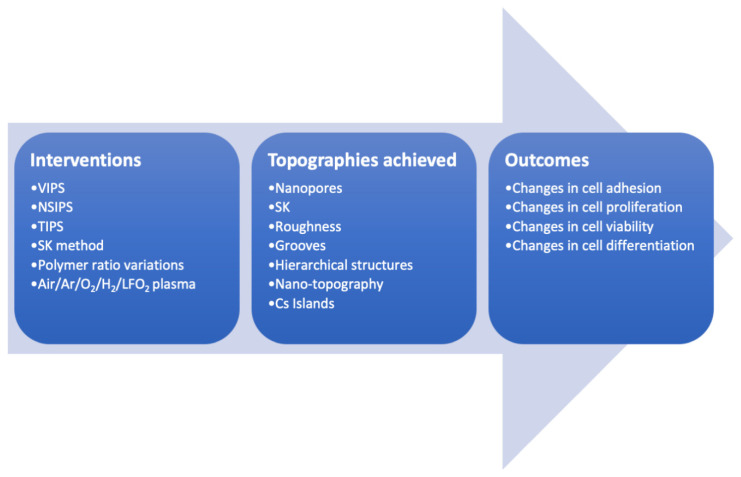
Possible interventions, achieved topographies and outcomes.

The results are summarized in Table 2 and Figure 2. The individual interventions presented in this table are explained in detail in the source papers. It is outside of the scope of this review to describe the interventions, since the analysis to be drawn is focused on the relation between the result of the interventions (topography) and cell–scaffold interactions (viability, adhesion, proliferation, differentiation).

Table 2 presents the studies grouped by the results of the intervention. The material used for the electrospun scaffold, how it was treated, the topographical features that results, which cells were used, and the outcome (improvement, worsening or equal tendencies of adhesion, proliferation, viability, or differentiation) are shown for each study. Additionally, within individual groupings in this table, studies are organized by risk of bias results. That is, studies that have less indicators of potential bias in the evaluation are on top. As the studies become less reliable, they are positioned more toward the bottom of their group. All abbreviations are explained at the bottom of the table.

At the top of the table, studies involving nanopores are grouped. In 2009, Leong et al. [35] used VIPS while electrospinning a poly-lactic acid (PLA) scaffold to induce nanopores on the fibers. After seeding the scaffolds with porcine esophageal epithelial cells, they found improved cell adhesion on the fibers with nanopores. Mertgen et al. [40] used VIPS to electrospin polycaprolactone (PCL) scaffolds into variations with more porous fibers. Although their control was also grooved, the proliferation of human umbilical vein endothelial cells (HUVECs) was significantly increased on the scaffolds with more defined topographical features. Other studies in this grouping fell into a lower bias group due to their evaluated risk of bias result.

The second group in the table includes studies involving Shish-Kebab structures (SKs+). In 2013, X. Wang et al. reported using an acetic acid/deionized water/PCL solution in order to induce SKs on a PCL scaffold [48]. An increasing concentration of the solution yielded larger and more defined kebabs. Cell viability and proliferation of murine 3T3 fibroblasts showed increased viability for the sample with the highest solution concentration applied to it. The proliferation assay did not result in significant differences. In 2019, Jing et al. [51] used a pentyl acetate/PCL solution to induce self-crystallization on PCL fibers, effectively creating SK structures on the electrospun scaffolds. They used this process on 2D and 3D scaffolds. The SK structures on the 2D scaffolds showed no significant differences in fibroblast proliferation when compared to the 2D control, but the 3D scaffolds with SKs showed more proliferation than the 3D control. In 2019, Yu et al. also used pentyl acetate to effectively form SKs on a PCL scaffold [53]. Murine MC3T3-E1 cells saw improving proliferation with SK incubation time and therefore SK size. Alkaline phosphatase (ALP) activity was also improved for most incubation times compared to a pristine PCL scaffold, especially at incubation times that resulted in a kebab periodicity on the order of collagen spacing. The other studies belonging to this group fell into a lower bias group.

The third grouping involved an increase of roughness on the fibers. This, however, was sometimes mixed with nanopore creation. In 2015, Zhou et al. [57] used VIPS to electrospin poly-L-lactic acid (PLLA) into rougher scaffolds, then used vascular smooth muscle cells (vSMCs) in order to evaluate their biocompatibility. VIPS, with increasing relative humidity, increased the roughness of the surface. Rougher surfaces led to improved adhesion and proliferation. These surfaces, at a higher level of roughness, could also be said to present nanopores.

Finally, there are some studies that added particular types of topography. Schaub et al., in 2013 [44], used non-solvent induced phase separation (NSIPS) in order to create nanoscale depressions (grooves) on PLLA fibers. RAW264.7 Macrophages did not show any significant differences other than cell morphology. Other topography types have been indeed studied, but those studies were evaluated to have a higher risk of bias in the previous section.

### 3.6. Risk of Bias across Studies

The results of the evaluation of risk of bias across studies are presented below, in Table 3, Table 4 and Table 5. These tables present the evidenced effect of the different topographical changes (Table 1, nanopores; Table 2, shish kebabs; Table 3, increased fiber roughness) and the associated quality of the evidence reviewed. The quality of the evidence associated to each of the topographical changes considered was found to be varied.

**Figure 3:**
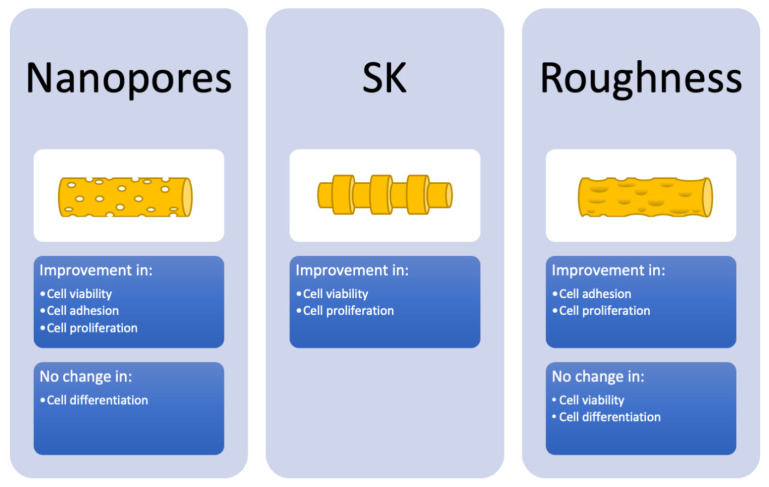
Schematic overview of the results of the GRADE tables.

Other topography types resulting from different interventions could not be included in the evaluation of risk of bias across studies, since they were only studied in one or two papers. The obtained results were visualized in Figure 3.

### 3.7. Risk of Bias in this Review

The evaluation of risk of bias in this review is presented in Supporting Information File S4. The main weakness identified through AMSTAR is the lack of duplicate study selection and data extraction.

## 4. Discussion

Before discussing the evidence presented in this review, a set of morphological and physicochemical variables that interact with fiber surface topography must be discussed, since these can contribute to risk of bias and therefore have a bearing on the applicability of the evidence as a whole. It is important to note that not all outcomes were evaluated by all studies.

Increased surface roughness generally has an effect on hydrophobicity [58], which itself has an effect on cell adhesion [59]. Particularly in a study by Schaub et al. [44], hydrophobicity was not affected by the topographical modification. If hydrophobicity changes were responsible for the improved cell–scaffold interactions, this could explain why this topographical modification saw no improved viability or adhesion.

Topographical features alter the pore adjacent to the fiber in varying measures. Altering the shape and size of this pore is known to affect cell–scaffold interactions [60,61]. Additionally, a relationship between the functionality of a scaffold and its specific surface area has previously been established [62]. Any addition of topographical features on a smooth fiber increases specific surface area.

Plasma modification, in general, is barely significant in this review since it implicitly incurs in performance bias, due to the intervention causing not only topographical, but also chemical modifications. It is important to note that the large amount of risk of bias attributed to the plasma modification studies [32,34,41,42,43,45,55,56] does not imply that these cannot establish a relationship between plasma application and a better cell–scaffold interaction, but rather, that these studies cannot establish a relationship between topographical modifications and a better cell–scaffold interaction. Therefore, it is outside of the scope of this review to quantify the extent to which the topographies elicited by plasma modification are responsible for that interaction. Such discussions can be found in other reviews that investigate the effect of topographies on cells [63].

On the other hand, shish-kebab (SK) modification studies [38,48,50,51,53] were mostly evaluated to have a low risk of bias, a symptom of the design of these studies. However, SKs, to a certain extent, cause performance bias, due to altering the general morphology of the scaffold by invading into the interfiber pores, thus affecting variables that are known to affect cell behavior. Another potential bias source for SKs is that the self-crystallization process potentially leads to more hydrophobic properties [64], potentially altering cell–scaffold interactions. It is currently hard to know whether SKs are beneficial to cell interaction due to the topography of the fiber surface, or they are beneficial to cell interaction due to their effect on the scaffold morphology (since they invade into the pore significantly more than other modifications such as nanopores). The relevance of these potential risks of bias seems non-transcendental to the result of this review: the topographical modification may well affect other variables, but whether the effect is direct or indirect, it affects cell–scaffold interactions.

Phase separation methods, similarly, mostly obtained better bias scores. This can be attributed to the design premise of these studies [33,35,37,38,39,40,44,47,52,54,57]. Something to account for, however, is indirectness, considering that different solvents and nonsolvents were used which have different evaporation rates. It is a known issue that residual solvent amounts in the final electrospun scaffold can have a cytotoxic effect [18,65]. The topographical features also differ significantly within this group.

Considering the objective of this review, it was coherent to present the results grouped by the results of the interventions, i.e., the topographical features elicited by the intervention, since the question asked is not necessarily the question that was answered by the data: whether topography improves cell–scaffold interactions may depend on what sort of topography it is. In this sense, the results of the risk of bias across studies evaluation are the ones that confer information about the completeness of the evidence. For example, some of the studies involve types of topography for which the available amount of data may be insufficient. These studies are the ones not included in the risk of bias across studies evaluation. It speaks to the incompleteness of the evidence and shows that this field still requires much research in order to ascertain what better surface morphology means.

As for applicability, the indirectness of the evidence both affects the applicability and the quality of the evidence in this review. By systematizing and structuring future research, it would be possible to reduce indirectness and improve the applicability and quality of evidence.

**Figure 4:**
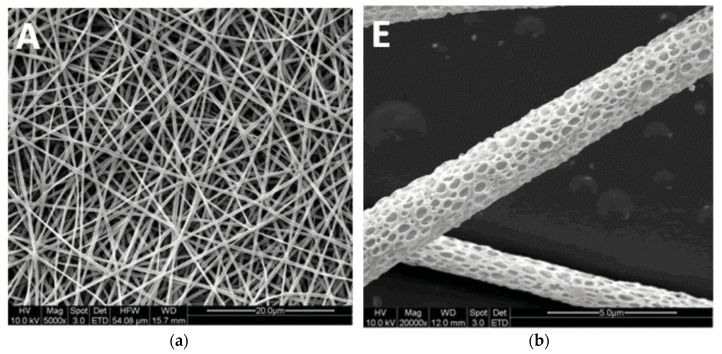
(**a**) Smooth fibers. (**b**) Nanoporous fibers. Samples B, D and F are not shown since they were coated or mineralized. The white letters at the top left of each image are the original labels. Reused from [37] with kind permission from John Wiley and Sons.

Firstly, the effect on viability of inducing fiber surface nanopores, visible in Figure 4, is only backed by highly biased studies [37,38,49], meaning that the evidence, although it points to a positive effect, cannot be trusted to be reliable for the purposes of this review. However, although somewhat scarce, the evidence for improved adhesion [35,47,57] and proliferation [37,38,39,40,47,49,54,57] was assessed to be minimally biased. Differentiation saw no major differences with nanopores, although the evidence for this is biased and extremely scarce [33,37].

**Figure 5:**
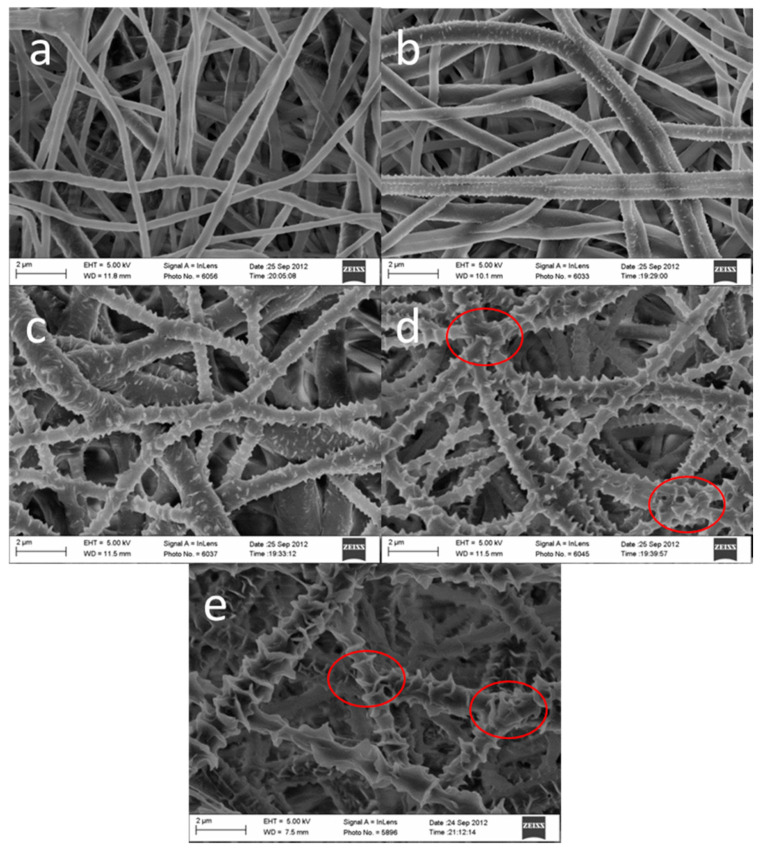
(**a**) Smooth electrospun fibers, and (**b**–**e**) fibers treated with increasing polymer concentrations. The red circles indicate fiber union points. Reprinted with permission from [48]. Copyright 2013 American Chemical Society.

For SK structures, which can be seen in Figure 5, the evidence mostly points to improved viability [38,48,50]. The quality of evidence for this is considered low. With some evidence pointing to improved cell proliferation and others seeing no significant difference, inconsistency was a factor in downgrading the quality of evidence for improved proliferation [38,48,50,51,53]. Adhesion and differentiation were not studied on SK structures.

**Figure 6:**
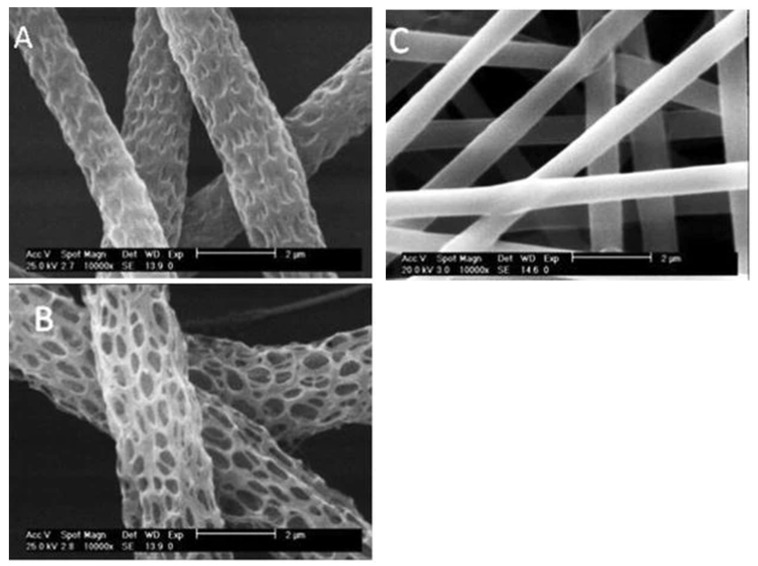
SEM images of (**A**) rough (grooved) fibers, (**B**) rougher (nanoporous) fibers (**C**) smooth fibers. Reused with kind permission from Springer Nature [54].

**Figure 7:**
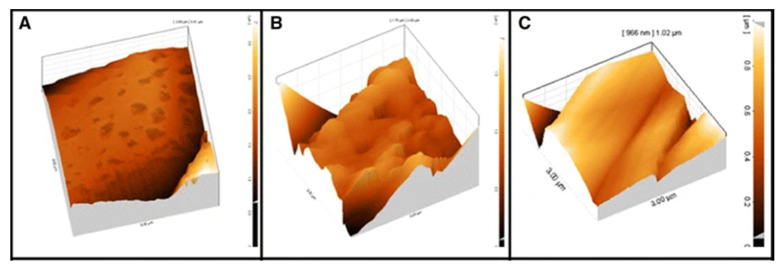
AFM images of (**A**) rough (grooved) fibers, (**B**) rougher (nanoporous) fibers (**C**) smooth fibers, from Figure 6. Reused with kind permission from Springer Nature [54].

For increased fiber surface roughness, which can be observed in Figure 6 and Figure 7, evidence points to viability not being significantly altered [32,33,43,45]. However, the quality of evidence makes this questionable. Somewhat better-quality evidence points to improved adhesion [34,56,57] and proliferation [34,41,42,45,54,57]. The evidence regarding differentiation [33,41] is extremely scarce and points to complex results, with the lowest risk of bias article [33] pointing to improved osteogenic differentiation, but no significant differences in chondrogenic differentiation.

Schaub et al. [44] achieved a grooved structure on their scaffolds, which showed no differences in viability or adhesion. All other studies [36,46,52,55], which involve various methods that contribute topographical features to otherwise smooth fibers, saw results tending toward improved proliferation, were divided regarding adhesion, and showed no significant impact on viability and differentiation. However, such a generalization is questionable considering the diversity, indirectness, and high risk of bias of the involved evidence.

The previous four paragraphs describe the most generalizable results because they involve the comparison of the results of the intervention (topography) and outcome (cell–scaffold interaction), and the risk of bias evaluation was done with this in mind. However, for the purposes of completeness, the next paragraph will evaluate the effect of material and cell type on the benefits of increased topographical features for cell–scaffold interactions. It is important to note that this is a highly biased approach and is only done for relationships that are clearly apparent so as to inform of the applicability of these results.

PCL scaffolds seem to benefit greatly from increased topographical features, since proliferation increased in almost every PCL scaffold that was modified. On all scaffolds with chitosan, topographical features resulted in improved cell–scaffold interactions. However, all studies that evaluated this were highly biased in the first place. All studies with endothelial cells saw improved cell–scaffold interactions when topographical features were added. Studies with fibroblasts mostly saw improved cell–scaffold interactions, whereas studies with stem cells only saw benefits in a minority of cases. Particularly, differentiation of stem cells was mostly unaffected by topography.

Risk of bias across studies was evaluated according to GRADE criteria. The number of papers using the same method is limited and the methods are often used very differently, or at least with varying parameters, often trying to posit highly multidimensional questions while controlling few variables. However, when regarding only the effect of particular topographies on cell–scaffold interactions, certain trends in the available literature can be described.

Since most evaluated studies did not attempt to infer the effect of topography on cell–scaffold interactions, but rather to infer the effect of the method (e.g., plasma) on cell–scaffold interactions, the studies did not fully align with this review’s established PICO criteria. Possibly due to this difference, most studies did not quantify the dimensions of the topographical features on the scaffold fibers. Of those that did, only one [54] thoroughly characterized the topography. Few reported either a periodicity/distance of the features, or an R_a_ value (the average height of the peaks and valleys of a roughness profile). This patent indirectness is the reason that a systematic review format was more appropriate than a meta-analysis, and it had an important effect on the risk of bias across studies evaluation, causing a downgrade in almost every single criterion.

Publication bias is an issue for all literature reviews. It was deemed inappropriate to perform a funnel plot analysis due to the inconsistencies in study design and indirectness of the studies. The lack of overwhelming evidence for the measured outcomes would offer room for cases in which topography affected cell–scaffold interactions negatively. A negative effect was reported only in one case [46], in which one of the scaffolds with more PEO content performed more poorly than the control. This, however, can barely be attributed to topography. Despite this, the authors claimed that nanotopography featured scaffolds improved proliferation. Other studies reported no negative effects at all. This might be due to publication bias in the form of lag bias, which results from more positive research being published earlier or faster, if at all. The fact that most studies are small and relatively recent increases the chances of lag bias being a factor in this review. Additionally, language bias can be an issue [66]. Since only reviews were chosen which were published in the English language, which generally has more circulation, journals in a different language, with smaller circulation, might have been chosen for articles with negative or inconclusive results. All in all, although some amount of publication bias is probable, it is worth noting that the Cochrane Handbook notes that publication bias is not the only explanation for these disparities, and that even a correctly made funnel plot can be insufficient for such a conclusion.

Deviations from the protocol were made that were deemed necessary to improve the review process. This included slightly changing the wording of PICO criteria, as well as adopting methods outlined by Cochrane or GRADE that were not originally included in the protocol. Additionally, the results were presented in tabular form, and the originally proposed narrative synthesis became a description of Table 2.

Publication status was an inclusion criterion. Two studies that were selected for full-text evaluation were not included due to them not being found. One of them was a conference presentation, as opposed to a research article, and the other appeared to be a false listing by the search engine. Further, two studies were excluded from the initial search that were not in the English language, and the “research article” filter on ScienceDirect allowed multiple review articles into the search results, potentially meaning that some research articles tagged as review articles might have been excluded.

The control criterium chosen was suboptimal, considering that in scaffolds with surface features induced by phase separation, there is no treatment to the scaffolds at all. They are simply created with different surface features. Therefore, the risk of bias in these studies might differ from the others. This is accounted for in the risk of bias evaluation, but it is difficult to understand the extent to which this is relevant.

The measurement methods and devices used to characterize scaffolds were not compared for their accuracy or reliability, and the methods to assess cell–scaffold interactions were also not compared.

PICO studies the effects of an intervention on an outcome, whereas this review intends to study the effects of multiple different interventions with a range of similar results on an outcome. Viability as an outcome was included later in the process. Regarding differentiation, progenitor cells can be differentiated into multiple different cell types, which might be differently affected by topography, meaning that differentiation might actually be a grouping of outcomes.

As can be seen in the AMSTAR evaluation, there are a few major sources of bias in this review, including the lack of a second person to evaluate studies. Publication bias, which can have a large bearing on the results, could not be quantitatively assessed. However, the quality of evidence was indeed downgraded to account for the possibility of it. It is important to note that it is an author judgment call to only downgrade evidence by one point for both indirectness and publication bias, and that this should be accounted for by users of this systematic review. This decision was taken accounting for the weight of each downgrading factor and the fact that the extent of publication bias could not be ascertained.

There are a few points that researchers could apply in order to systematize research in this field. These include, when possible, always making concrete measurements of all topographic properties of the fibers. For example, the distances between topographic features as well as the height of these topographic features, or a roughness profile measurement. Measuring the fiber diameter could be standard practice, as well as the average pore size and porosity of the scaffold. If these measurements are made on future topographically modified scaffolds, a meta-analysis to evaluate their effect might become viable. However, it must also be said that, even if all these factors are measured, scaffolds made with different materials, methods, and seeded with different cells might make the comparison questionable. It is probable that much research must be made, or perhaps large, systematic studies, before any statistically sound comparisons can be made.

In future work, it would be interesting to replicate studies carried out with films to see if 3D structures elicit the same effects when modifying topography. Additionally, there is a need to explore dose dependency. How the magnitude of topographic features affects cell–scaffold interactions remains to be seen. This is something that should be studied for specific cell types and specific polymers and might provide insights into the mechanisms of action underlying the effects reported in this review. Such an analysis could be conducted with the help of computational modeling. It would also be interesting to compare some of the interventions discussed in this review.

## 5. Conclusions

Readers should be warned of spurious correlations within similar materials, interventions, or cell types, especially within the same intervention method (e.g., plasma, phase separation). Extracting conclusions from these groupings involves too much risk of bias that has not been accounted for. On the other hand, results of interventions can be more sensibly compared since the performed risk of bias evaluation is specifically related to effects of the dimensions of the topography. This topography was, unfortunately, not sufficiently characterized by most studies included, making a meta-analysis to this end non-viable. Additionally, the differing materials and the lack of exact quantification of materials’ effect on cell–scaffold interactions would have caused a large amount of potential bias in such a quantitative approach.

The conclusions that can be taken from the analyzed data in this review are the following. The quality of evidence (QoE) is presented along with the results. Articles with nanopores on fibers, as evaluated in the present review, reported improved viability (low QoE), adhesion (moderate QoE), and proliferation (high QoE) when compared to smoother fibers. Shish-kebab structures, in the evaluated studies, led to improved viability (low QoE) and proliferation (moderate QoE). Increased fiber roughness, in the included articles, led to improved adhesion (low QoE) and proliferation (moderate QoE). All other results presented a QoE that was very low.

It is of essence to note that the lack of large, systematically designed studies implies a low quality of evidence. This is furthered by the indirectness of the involved studies. It is therefore our assessment that this question, although it shows promising trends so far, must be studied in a more quantitative manner in the future so as to improve the quality of evidence available and thus permit a meta-analysis. An investigation into dose-dependency relationships could clarify how the magnitude of topographic features affects cell–scaffold interactions.

The research included in this field, if continued, might provide insight into the role of topography in cell–scaffold interactions. It would be optimal to control these interactions for specific cell types and materials. If topographical cues provide a solution to this conundrum, or even an improvement, then researchers might have a financially and technologically accessible method to optimize their scaffolds for the cell line they desire.

## Figures and Tables

**Table 1:** Risk of bias within studies as evaluated.

	Was There a Clear Statement of the Aims of the Research?	Was the Methodology Appropriate to Address the Aims of the Research?	Is There an Untreated Electrospun Control?	Was the Data Analysis Method for Cell–Scaffold Interactions Reported?	No Selection Bias	No Performance Bias (i.e., Were There Other Changes Made to the Scaffolds?)	No Detection Bias (i.e., Does the Modification Affect Measurements?)	No Reporting Bias (i.e., Selective Outcome Reporting)	Is There a Clear Statement of Findings?	No Other Apparent Bias Sources
**Correia 2016 [32]**	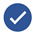	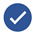	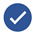	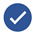	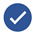	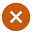	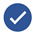	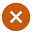	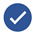	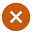
**Chen 2017 [33]**	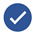	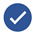	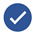	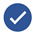	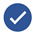	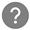	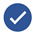	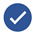	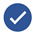	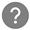
**Jeon 2014 [34]**	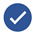	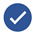	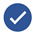	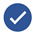	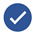	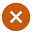	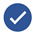	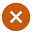	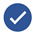	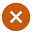
**Leong 2009 [35]**	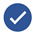	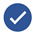	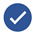	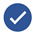	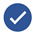	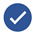	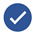	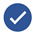	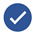	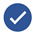
**Li 2014** [36]	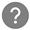	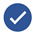	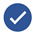	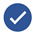	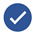	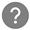	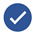	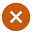	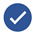	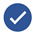
**Liao 2014 [37]**	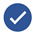	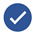	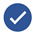	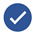	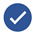	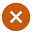	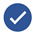	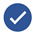	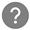	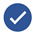
**Jiang 2018 [38]**	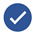	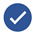	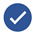	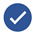	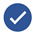	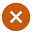	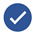	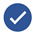	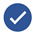	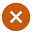
**Moroni 2006 [39]**	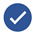	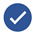	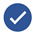	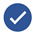	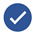	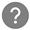	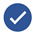	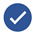	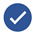	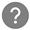
**Mertgen 2018 [40]**	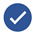	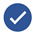	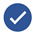	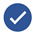	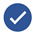	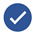	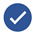	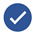	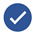	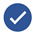
**Nandakumar 2013** [41]	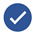	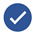	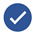	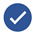	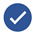	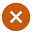	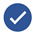	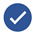	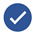	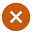
**Ozkan 2016 [42]**	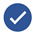	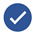	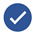	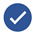	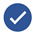	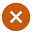	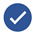	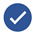	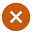	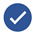
**Das 2018 [43]**	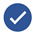	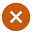	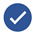	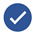	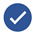	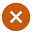	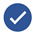	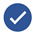	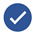	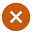
**Schaub 2013 [44]**	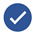	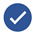	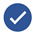	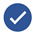	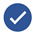	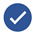	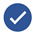	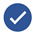	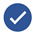	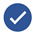
**Surucu 2016 [45]**	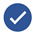	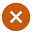	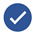	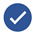	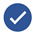	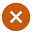	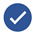	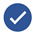	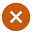	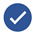
**Taskin 2017 [46]**	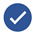	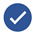	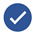	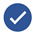	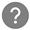	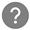	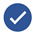	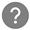	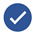	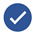
**Wang, T. 2013 [47]**	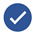	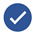	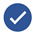	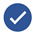	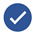	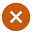	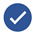	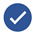	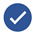	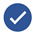
**Wang, X. 2013 [48]**	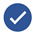	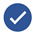	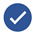	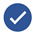	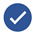	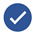	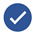	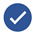	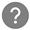	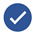
**Wang, Y. 2015 [49]**	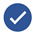	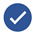	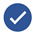	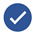	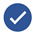	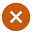	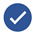	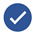	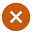	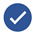
**Jing 2014 [50]**	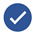	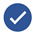	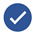	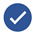	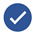	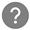	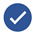	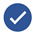	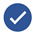	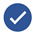
**Jing 2019 [51]**	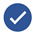	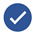	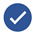	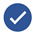	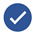	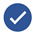	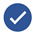	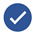	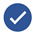	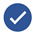
**Xu 2017 [52]**	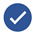	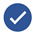	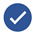	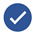	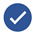	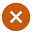	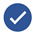	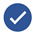	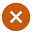	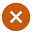
**Yu 2019 [53]**	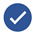	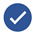	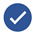	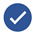	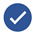	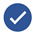	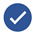	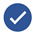	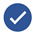	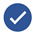
**Zamani 2013 [54]**	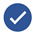	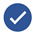	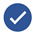	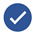	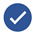	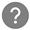	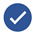	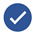	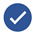	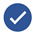
**Zandén 2014 [55]**	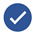	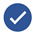	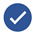	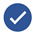	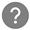	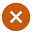	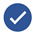	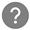	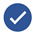	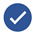
**Zandén 2012 [56]**	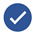	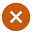	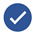	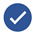	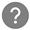	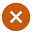	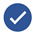	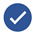	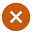	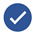
**Zhou 2015 [57]**	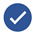	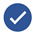	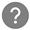	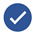	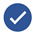	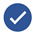	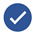	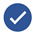	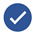	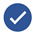

Symbol meaning: 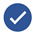 yes, 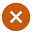 no, 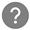 unclear.

**Table 2:** Presentation of results.

Ref.	Electrospun Material	Intervention	Results of Intervention	Cells	Cell-Intervention Interaction	BG
[35]	PLA	VIPS	Nanopores	PEECs	Adh +	1
[40]	PCL	VIPS	Nanopores	HUVECs	Prol +	1
[39]	PEOT/PBT	NSIPS	Nanopores	hMSCs	Prol +	2
[47]	PLLA (+Coll.)	NSIPS	Nanopores	PRHs	Adh =, Prol + (↓)	3
[37]	PLLA	NSIPS	Nanopores	hMSCs	Viab =, prol =, diff =	3
[49]	PPC/PCL	PCL ratio variations	Nanopores	Adipose cells	Viab+, Prol =	4
[38]	PCL	VIPS & SK	Nanopores & SKs	3T3MFs	Viab +, Prol +	4
[38]	PCL	VIPS & SK	Nanopores & SKs	HUVECs	SKs: Viab +. Prol + NPs: Viab ++, Prol++	4
[48]	PCL	SK	SKs +	3T3MFs	Viab +, Prol =	1
[51]	PCL	2D/3D SKs	SKs +	3T3MFs & HFs	2D: Prol = 3D: Prol +	1
[53]	PCL	SKs	SKs +	MC3T3-E1	Prol +	1
[50]	PCL	SK	SKs +	HEF1s	Viab =, Prol +	2
[57]	PLLA	VIPS	Roughness + & Nanopores	vSMCs	Adh +, Prol +	1
[54]	PLGA	VIPS, NSIPS Polymer ratio	Roughness + & Nanopores	A-172	Prol +	2
[33]	PEOT/PBT	VIPS	Roughness +	hMSCs	Viab =, ost. diff + Chond. Diff =	2
[56]	PU	O_2_ plasma	Roughness +	RBCs	Adh = (↓)	3
[41]	PEOT/PBT	O_2_ plasma	Roughness +	hMSCs	Prol =, Diff +	3
[34]	PCL	LFO_2_ plasma	Roughness +	MG63OCs	Adh +, Prol +	4
[32]	PLLA	O_2_ plasma	Roughness +	MC3T3-E1	Viab =	4
[42]	PCL/Cs	Air plasma	Roughness +	MFs	Prol+	4
[43]	PVAC/Cs	DBD O_2_, Ar plasma	Ar: Roughness + O_2_: Roughness ++	MFs	Ar: Viab + (↓) O_2_: Viab ++ (↓)	4
[45]	PCL/Cs	Ar, air plasma	Ar: Roughness + Air: flat fibers	MRC5 HFs	Ar: Viab =, Prol + Air: Viab +, Prol +	4
[44]	PLLA	NSIPS	Grooves +	RAW264.7	Viab =, Adh=	1
[36]	PCL/PEO	PEO ratio variations	Hierarchical structures	RAW264.7	Viab =, Adh=	3
[46]	PCL/PEO	PEO ratio variations	Nano-topography	HUVECs	Adh +, Prol +	3
[55]	PU	O_2_, Ar, H_2_ plasma	Topography variations	SA121 hESCs	O_2_, H_2_ RONs: Prol+ Ar RONs: Prol ++ ANs: Prol = Diff =	4
[55]	PU	O_2_, Ar, H_2_ plasma	Topography variations	RNSCs	Prol =, Diff =	4
[52]	PLA/Cs	TIPS	Cs Islands	POMCs	Prol +, Diff=	4

Abbreviations: +: results improved compared to control, ++: results improved compared to (+) sample. =: Results show no significant difference compared to control/comparator. ↓: Even if this sample was better than the control, it still showed decaying or low values. A-172: A line of brain glioblastoma cells. Adh: Adhesion. BG: Bias groups 1–4. Group 1 has the least bias in relating intervention results to outcome. Chond.: Chondrogenic. Coll.: Collagen. Cs: Chitosan. Diff: Differentiation. HEF1s: HEF1 fibroblast cells differentiated from hESCs. hESCs: human embryonic stem cells. HFs: Human fibroblasts. hMSCs: Human mesenchymal stem cells. HUVECs: human umbilical vein endothelial cells. MC3T3-E1: Mouse osteblastic cells. MFs or 3T3MFs: Mouse fibroblasts. MG63OCs: MG63 line osteosarcoma cells. NSIPS: (Non-)Solvent induced phase separation. Ost.: Osteogenic. PCL: Polycaprolactone. PEECs: Porcine esophageal endothelial cells. PEO: Polyethylene oxide. PEOT/PBT: poly (ethylene oxide terephthalate)/poly (butylene terephthalate). PLA: Polylactic acid. PLGA: poly (lactic-*co*-glycolic acid). PLLA: poly-L-lactic acid. POMCs: Preosteoblastic mouse cells. PPC: Polypropylene carbonate. PRHs: Primary rat hepatocytes. Prol: Proliferation. PU: Polyurethane. PVAC: Polyvinyl acetate. RAW264.7: A line of macrophages. RBCs: Red blood cells. RNSCs: Rodent neural stem cells. SK: Shish-kebab. TIPS: Thermally induced phase separation. Viab: Viability. VIPS: Vapor induced phase separation. vSMCs: vascular smooth muscle cells.

**Table 3:** Risk of bias across studies for nanopores on fibers compared to no nanopores on fibers for cells on electrospun scaffolds.

Outcome	Number of Studies	Quality of Evidence (GRADE)	Anticipated Effect
**Viability**	**3** [37,38,49]	(+) (−) (−) (−) **LOW** Due to study−level bias, indirectness ^1^, imprecision	Improvement
**Adhesion**	**3** [35,47,57]	(+) (−) (−) **MODERATE** Due to indirectness ^1^, imprecision	Improvement
**Proliferation**	**8** [37,38,39,40,47,49,54,57]	(+) (−) **HIGH** Due to indirectness ^1^	Improvement
**Differentiation**	**1** [33,37]	(−) (−) (−) **VERY LOW** Due to study−level bias, indirectness ^1^, imprecision	No Change

Quality of evidence grades: High: We are very confident that the true effect lies close to that of the estimate of the effect. Moderate: We are moderately confident in the effect estimate: The true effect is likely to be close to the estimate of the effect, but there is a possibility that it is substantially different. Low: Our confidence in the effect estimate is limited: The true effect may be substantially different from the estimate of the effect. Very Low: We have very little confidence in the effect estimate: The true effect is likely to be substantially different from the estimate of effect. ^1^ Since both indirectness and publication bias were a constant in all included studies, they were only downgraded once to account for both matters.

**Table 4:** Risk of bias across studies for studies involving shish kebabs compared to no shish kebabs for cells on electrospun scaffolds.

Outcome	Number of Studies	Quality of Evidence (GRADE)	Anticipated Effect
**Viability**	[38,48,50]	(+) (−) (−) (−) **LOW** Due to study−level bias, indirectness ^1^, imprecision	Improvement
**Adhesion**	**0**	**N/A**	**N/A**
**Proliferation**	**5** [38,48,50,51,53]	(+) (−) (−) **MODERATE** Due to inconsistency, indirectness ^1^	Improvement
**Differentiation**	**0**	**N/A**	**N/A**

Quality of evidence grades: High: We are very confident that the true effect lies close to that of the estimate of the effect. Moderate: We are moderately confident in the effect estimate: The true effect is likely to be close to the estimate of the effect, but there is a possibility that it is substantially different. Low: Our confidence in the effect estimate is limited: The true effect may be substantially different from the estimate of the effect. Very Low: We have very little confidence in the effect estimate: The true effect is likely to be substantially different from the estimate of effect. ^1^ Since both indirectness and publication bias were a constant in all included studies, they were only downgraded once to account for both matters.

**Table 5:** Risk of bias across studies for studies involving increased fiber roughness compared to no increased fiber roughness for cells on electrospun scaffolds.

Outcome	Number of Studies	Quality of Evidence (GRADE)	Anticipated Effect
**Viability**	**4** [32,33,43,45]	(+) (−) (−) (−) (−) **VERY LOW** Due to study−level bias, inconsistency, indirectness ^1^, imprecision	No Change
**Adhesion**	**3** [34,56,57]	(+) (−) (−) (−) **LOW** Due to study−level bias, indirectness ^1^, imprecision	Improvement
**Proliferation**	**6** [34,41,42,45,54,57]	(+) (−) (−) **MODERATE** Due to study−level bias, indirectness ^1^	Improvement
**Differentiation**	**2** [33,41]	(−) (−) (−) (−) **VERY LOW** Due to study−level bias, inconsistency, indirectness ^1^, imprecision	No Change

Quality of evidence grades: High: We are very confident that the true effect lies close to that of the estimate of the effect. Moderate: We are moderately confident in the effect estimate: The true effect is likely to be close to the estimate of the effect, but there is a possibility that it is substantially different. Low: Our confidence in the effect estimate is limited: The true effect may be substantially different from the estimate of the effect. Very Low: We have very little confidence in the effect estimate: The true effect is likely to be substantially different from the estimate of effect. ^1^ Since both indirectness and publication bias were a constant in all included studies, they were only downgraded once to account for both matters.

## Data Availability

A protocol for this systematic review is available online at https://figshare.com/articles/online_resource/Review_Protocol_Fiber_Surface_Properties_docx/13523822/1 (accessed on 30 November 2021).

## Supplementary Materials

The following supporting information can be downloaded online at https://www.mdpi.com/article/10.3390/polym14010209/s1. Supplementary Information File S1: Justification for excluded articles. Supplementary Information File S2: Designed data extraction form. Supplementary Information File S3: Filled data extraction forms. Supplementary Information File S4: AMSTAR checklist.

## Abbreviations

A-172	A line of brain glioblastoma cells
Adh	Adhesion
BG	Bias groups 1-4 (group 1 having the lowest bias level)
Chond.	Chondrogenic
Coll.	Collagen
Cs	Chitosan
Diff	Differentiation
HEF1s	HEF1 fibroblast cells differentiated from hESCs
hESCs	human embryonic stem cells
HFs	Human fibroblasts
hMSCs	Human mesenchymal stem cells
HUVECs	human umbilical vein endothelial cells
MC3T3-E1	Mouse osteblastic cells
MFs or 3T3MFs	Mouse fibroblasts
MG63OCs	MG63 line osteosarcoma cells
NSIPS	(Non-)Solvent induced phase separation
Ost.	Osteogenic
PCL	Polycaprolactone
PEECs	Porcine esophageal endothelial cells
PEO	Polyethylene oxide
PEOT/PBT	poly (ethylene oxide terephthalate)/poly (butylene terephthalate)
PLA	Polylactic acid
PLGA	poly (lactic-co-glycolic acid)
PLLA	poly-L-lactic acid
POMCs	Preosteoblastic mouse cells
PPC	Polypropylene carbonate
PRHs	Primary rat hepatocytes
Prol	Proliferation
PU	Polyurethane
PVA	Polyvinyl acetate
RAW264.7	A line of macrophages
RBCs	Red blood cells
RNSCs	Rodent neural stem cells
SK	Shish-kebab
TIPS	Thermally induced phase separation
Viab	Viability
VIPS	Vapor induced phase separation
vSMCs	vascular smooth muscle cells
