# Supplementary material for: How Fiber Surface Topography Affects Interactions between Cells and Electrospun Scaffolds: A Systematic Review
Source: Polymers (Basel). 2022 Jan 5;14(1):209. doi: 10.3390/polym14010209 (PMC8747153; doi:10.3390/polym14010209)
Supplement: Supplementary file 1 [file polymers-14-00209-s001.zip › Supplementary Information File S1.pdf]

Author(s)	Title	Periodical	DOI name	Notes/Justification
Abdolrahman Omidir	Hierarchical fibrous guiding cues at different scales influence linear neurite extension	Acta biomaterialia	10.1016/j.actbio.2020.07.014	Not electrospinning but Solvent assisted spinning
Aidun, A.; Zamanian,	Immobilization of polyvinyl alcohol-siloxane on the oxygen plasma-modified polyurethane-carbon nanotube composite matrix	Journal of Applied Pc	10.1002/app.48477	CNT use does not allow for isolation of topographical effects
Arabi, A.; Boggs, E.; Pa	Surface modification of electrospun polycaprolactone fibers and effect on cell proliferation	Surface Innovations	10.1680/si.13.00018	Topography not modified
C. Simitzi; P. Efstathop	Laser fabricated discontinuous anisotropic microconical substrates as a new model scaffold to control the directionality of neuronal network outgrowth	Biomaterials	10.1016/j.biomaterials.2015.07.008	Not electrospun
Carl Zandén; Marina	Surface characterisation of oxygen plasma treated electrospun polyurethane fibres and their interaction with red blood cells	European Polymer Jo	10.1016/j.eurpolymj.2012.01.004	Plasma modification caused surface texture change but red blood cells had similar behavior in control
Cheng, Q.; Lee, B.L.-P	Plasma surface chemical treatment of electrospun poly(L-lactide) microfibrous scaffolds for enhanced cell adhesion, growth, and infiltration	Tissue Engineering -	10.1089/ten.tea.2011.0725	No morphology modification
Correia, D. M.; Ribeiro	Superhydrophilic poly(L-lactic acid) electrospun membranes for biomedical applications obtained by argon and oxygen plasma	Applied Surface Scie	10.1016/j.apsusc.2016.02.121	Only Ra is accounted for, but it changed plasma treatment didn't affect cell viability much
Criscenti, G.; Vasilevic	3D screening device for the evaluation of cell response to different electrospun microtopographies	Acta biomaterialia	10.1016/j.actbio.2017.03.049	No surface topography modification, but shaped wells
D. Angelaki; P. Kavatz	Laser-induced topographies enable the spatial patterning of co-cultured peripheral nervous system cells	Materials Science and	10.1016/j.msec.2020.111144	Not electrospun, Si used
Dawei Li; Tong Wu; N	Three-dimensional polycaprolactone scaffold via needleless electrospinning promotes cell proliferation and infiltration	Colloids and Surfaces	10.1016/j.colsurfb.2014.06.034	Hard to isolate effect of pores in fibers: morphology is generally different in DE scaffolds
Elizabeth J. Tocce; Va	The ability of corneal epithelial cells to recognize high aspect ratio nanostructures	Biomaterials	10.1016/j.biomaterials.2010.01.101	Investigates contact guidance as opposed to cell adhesion, proliferation or differentiation
F. Chen; C.N Lee; S.H	Nanofibrous modification on ultra-thin poly(e-caprolactone) membrane via electrospinning	Materials Science and	10.1016/j.msec.2006.05.004	A film (membrane), not a scaffold, was coated with electrospun nanofibers
Fatemeh Jahanmard;	Incorporation of F-MWCNTs into electrospun nanofibers regulates osteogenesis through stiffness and nanotopography	Materials Science and	10.1016/j.msec.2019.110163	CNT use does not allow for isolation of topographical effects
Fei Xu; Ian Gough; Jor	Nanostructured degradable macroporous hydrogel scaffolds with controllable internal morphologies via reactive electrospinni	Acta biomaterialia	10.1016/j.actbio.2019.12.038	Multiple alterations made to the scaffolds no control used
Guzman, R. C. de; Loe	Electrospinning of matrigel to deposit a basal lamina-like nanofiber surface	Journal of Biomateria	10.1163/092050609X12457428936116	No relevant intervention
Hem Raj Pant; Madha	Fabrication of highly porous poly (ε-caprolactone) fibers for novel tissue scaffold via water-bath electrospinning	Colloids and Surfaces	10.1016/j.colsurfb.2011.07.045	No relevant intervention
Hong Nam Kim; Yoon	Effect of orientation and density of nanotopography in dermal wound healing	Biomaterials	10.1016/j.biomaterials.2012.08.038	No electrospinning involved
Honglin Chen; Xiaobi	Tailoring surface nanoroughness of electrospun scaffolds for skeletal tissue engineering	Acta biomaterialia	10.1016/j.actbio.2017.07.003	Cell differentiation is affected by controlling surface roughness
Huaqiong Li; Feng We	Direct laser machining-induced topographic pattern promotes up-regulation of myogenic markers in human mesenchymal ster	Acta biomaterialia	10.1016/j.actbio.2011.09.029	Substrate is a film, not electrospun
Hyun Jong Lee; Seung	Micropatterned Fibrous Scaffolds Fabricated Using Electrospinning and Hydrogel Lithography: New Platforms to Create Cellula	Sensors and Actuato	10.1016/j.snb.2010.05.032	Patterns are made on the scaffold, not individual fibers
J. Idaszek; E. Kijeńska;	How important are scaffolds and their surface properties in regenerative medicine	Applied Surface Scie	10.1016/j.apsusc.2016.03.038	This is a review
J. Pelipenko; P. Kocbe	The topography of electrospun nanofibers and its impact on the growth and mobility of keratinocytes	European Journal of	10.1016/j.ejpb.2012.09.009	Control is not an electrospun scaffold
Jaba Mitra; Shilpee Ja	Patterned growth and differentiation of neural cells on polymer derived carbon substrates with micro/nano structures in vitro	Carbon	10.1016/j.carbon.2013.08.008	Control is a film, not an electrospun substrate
Jaiswal, A.	Nanofibrous Scaffolds for Tissue Engineering Applications	Brazilian Archives of	10.1590/1678-4324-2016150644	Topography of scaffold as a whole, not fibers, was evaluated
James M. Dugan; Rich	Oriented surfaces of adsorbed cellulose nanowhiskers promote skeletal muscle myogenesis	Acta biomaterialia	10.1016/j.actbio.2012.08.050	Substrate not electrospun
Jeon, H.; Kim, G.	Preparation and characterization of an electrospun polycaprolactone (PCL) fibrous mat and multi-layered PCL scaffolds having a nanosized pattern-surface for tissue regeneration	Journal of Materials (	10.1039/c3tb21230k	Plasma treatment changes cell behavior
Jeong In Kim; Cheol S	Harnessing nanotopography of PCL/collagen nanocomposite membrane and changes in cell morphology coordinated with wound healing activity	Materials Science and	10.1016/j.msec.2018.06.021	Topography refers to fiber alignment here
Jie Shi Chua; Choon-P	Extending neurites sense the depth of the underlying topography during neuronal differentiation and contact guidance	Biomaterials	10.1016/j.biomaterials.2014.06.008	Substrate not electrospun
Johnson, C. D.; D'Ama	Electrospun fiber surface nanotopography influences astrocyte-mediated neurite outgrowth	Biomedical Materials	10.1088/1748-605X/aac4de	Outcome is neurite outgrowth, so not included

K.M. Pawelec; C. Yoor	**Engineering a platform for nerve regeneration with direct application to nerve repair technology**	Biomaterials	10.1016/j.biomaterials.2019.119263	Channeled scaffold was created, not with fiber surface topography
Kim, H. H.; Kim, M. J.;	**Effect of fiber diameter on surface morphology, mechanical property, and cell behavior of electrospun poly(ε-caprolactone) mat**	Fibers and Polymers	10.1007/s12221-016-6350-x	Fiber diameter was altered, also no proper control
Kim, J. I.; Hwang, T. I.;	**Regulating Electrical Cue and Mechanotransduction in Topological Gradient Structure Modulated Piezoelectric Scaffolds to Predict Neural Cell Response**	Advanced Functional	10.1002/adfm.201907330	Treats topography as fiber alignment/structure morphology
Leong, Meng Fatt; Ch	**Effect of electrospun poly(D,L-lactide) fibrous scaffold with nanoporous surface on attachment of porcine esophageal epithelial cells and protein adsorption**	Journal of Biomedica	10.1002/jbm.a.32061	
Lesley Y. Chan; Williar	**Temporal application of topography to increase the rate of neural differentiation from human pluripotent stem cells**	Biomaterials	10.1016/j.biomaterials.2012.09.033	Substrate not electrospun
Li, Yan-Fang; Rubert,	**Ultraporous interweaving electrospun microfibers from PCL–PEO binary blends and their inflammatory responses**	Nanoscale	10.1039/c3nr06197c	
Liao, Susan; Nguyen,	**Biomimetic Nanocomposites to Control Osteogenic Differentiation of Human Mesenchymal Stem Cells**	Advanced Healthcare	10.1002/adhm.201300207	
Lim, M. M.; Sun, T.; Su	**In vitro biological evaluation of electrospun polycaprolactone/gelatine nanofibrous scaffold for tissue engineering**	Journal of Nanomate	10.1155/2015/303426	Surface topography of fibers not regarded
Lin Jiang; Liwei Wang	**Fabrication of polycaprolactone electrospun fibers with different hierarchical structures mimicking collagen fibrils for tissue engineering scaffolds**	Applied Surface Scie	10.1016/j.apsusc.2017.08.005	
Lorenzo Moroni; Ruu	**Fiber diameter and texture of electrospun PEOT/PBT scaffolds influence human mesenchymal stem cell proliferation and morphology, and the release of incorporated compounds**	Biomaterials	10.1016/j.biomaterials.2006.05.027	
M. Tallawi; D. Dippolo	**Novel PGS/PCL electrospun fiber mats with patterned topographical features for cardiac patch applications**	Materials Science and	10.1016/j.msec.2016.06.083	topography of scaffold, not fibers, modified
M.M. Machado-Paula	**A comparison between electrospinning and rotary-jet spinning to produce PCL fibers with low bacteria colonization**	Materials Science and	10.1016/j.msec.2020.110706	Fibers are not modified, no cell assay
Maharjan, Bikendra; K	**In-situ polymerized polypyrrole nanoparticles immobilized poly(ε-caprolactone) electrospun conductive scaffolds for bone tissue engineering**	Materials science & e	10.1016/j.msec.2020.111056	Polypyrrole alters conductivity, does not isolate topography
Mertgen, A. S.; Yazga	**Controlling the surface structure of electrospun fibers: Effect on endothelial cells and blood coagulation**	Biointerphases	10.1116/1.5047668	
Miji Yeo; GeunHyung	**Nano/microscale topographically designed alginate/PCL scaffolds for inducing myoblast alignment and myogenic differentiation**	Carbohydrate Polym	10.1016/j.carbpol.2019.115041	Substrate not electrospun
Mirakabad, F. S. T.; Kh	**The effect of clay nanoparticles on surface topography of polymeric scaffolds and mesenchymal stem cell attachment**	BioImpacts	10.15171/bi.2018.S1	Clay use does not allow for isolation of topographical effects
Monireh Kouhi; Moha	**Poly L lysine-modified PHBV based nanofibrous scaffolds for bone cell mineralization and osteogenic differentiation**	Applied Surface Scie	10.1016/j.apsusc.2018.06.239	Roughness of scaffold, not fiber surface, modified
Nandakumar, A.; Birg	**Surface modification of electrospun fibre meshes by oxygen plasma for bone regeneration**	Biofabrication	10.1088/1758-5082/5/1/015006	some osteogenic markers were upregulated
Negar Abbasi Aval; Ra	**Nano-featured poly (lactide-co-glycolide)-graphene microribbons as a promising substrate for nerve tissue engineering**	Composites Part B: E	10.1016/j.compositesb.2019.05.074	Graphene use does not allow for isolation of topographical effects
Ozan Ozkan; Hilal Tur	**Effects of nozzle type atmospheric dry air plasma on L929 fibroblast cells hybrid poly (ε-caprolactone)/chitosan/poly (ε-caprolactone) scaffolds interactions**	Journal of Bioscience	10.1016/j.jbiosc.2016.01.004	
Park, B. J.; Seo, H. J.; K	**Cellular responses of vascular endothelial cells on surface modified polyurethane films grafted electrospun PLGA fiber with microwave-induced plasma at atmospheric pressure**	Surface & Coatings T	10.1016/j.surfcoat.2010.07.087	Substrate not electrospun
Punamshree Das; Nar	**Surface modification of electrospun PVA/chitosan nanofibers by dielectric barrier discharge plasma at atmospheric pressure and studies of their mechanical properties and biocompatibility**	International Journal	10.1016/j.ijbiomac.2018.03.115	
Roya Dastjerdi; Mahn	**A pathway toward new era of intelligent cell attachment; mechanism and a key major guideline**	Journal of Cleaner Pr	10.1016/j.jclepro.2020.121873	No control
Sachot, N.; Castaño, C	**Hierarchically engineered fibrous scaffolds for bone regeneration**	J. R. Soc. Interface	10.1098/rsif.2013.0684	
Saeed, M.; Mirzadeh,	**Rationalization of specific structure formation in electrospinning process: Study on nano-fibrous PCL- and PLGA-based scaffolds**	Journal of Biomedica	10.1002/jbm.a.35520	Varies scaffold morphology, not fiber surface
Saravana Kumar Jaga	**Biomimetic electrospun polyurethane matrix composites with tailor made properties for bone tissue engineering scaffolds**	Polymer Testing	10.1016/j.polymertesting.2019.105955	No modification of fiber surface
Schaub, N. J.; Britton,	**Engineered nanotopography on electrospun PLLA microfibers modifies RAW 264.7 cell response**	ACS applied material	10.1021/am402827g	
Seda Surucu; Kai Mas	**Atmospheric plasma surface modifications of electrospun PCL/chitosan/PCL hybrid scaffolds by nozzle type plasma jets for usage of cell cultivation**	Applied Surface Scie	10.1016/j.apsusc.2016.05.123	
Taskin, Mehmet Berat	**Nanotopography featured polycaprolactone/polyethyleneoxide microfibers modulate endothelial cell response**	Nanoscale	10.1039/C7NR03326E	
Tiryaki, V.; Ayres, V. M	**Quantitative investigation of differences in differentiation of reactive and nonreactive astrocytes in response to nanophysical cues**	Mol. Biol. Cell		Source appears to be a congress presentation, not accessible

Wang, Ting; Feng, Zh	**Nanoporous fibers of type-I collagen coated poly(l -lactic acid) for enhancing primary hepatocyte growth and function**	J. Mater. Chem. B	10.1039/C2TB00195K	
Wang, Xiaofeng; Salic	**Poly(ε-caprolactone) Nanofibers with a Self-Induced Nanohybrid Shish-Kebab Structure Mimicking Collagen Fibrils**	Biomacromolecules	10.1021/bm400928b	
Wang, Y.; Deng, J.; Fa	**Novel nanoscale topography on poly(propylene carbonate)/poly(ε-caprolactone) electrospun nanofibers modifies osteogenic capacity of ADCs**	RSC Advances	10.1039/c5ra15841a	
Xiaozhi Ren; Jinxiu Li;	**Aligned porous fibrous membrane with a biomimetic surface to accelerate cartilage regeneration**	Chemical Engineering	10.1016/j.cej.2019.03.271	Modification made by surface coating, all tested scaffolds are nanoporous, i.e. no control
Xin Jing; Hao-Yang M	**Fabrication of shish–kebab structured poly(ε-caprolactone) electrospun nanofibers that mimic collagen fibrils: Effect of solvents and matrigel functionalization**	Polymer	10.1016/j.polymer.2014.08.061	
Xin Jing; Heng Li; Hac	**Fabrication of fluffy shish-kebab structured nanofibers by electrospinning, CO2 escaping foaming and controlled crystallization for biomimetic tissue engineering scaffolds**	Chemical Engineering	10.1016/j.cej.2019.04.194	
Xu, Ting; Yang, Hong	**Polylactic Acid Nanofiber Scaffold Decorated with Chitosan Islandlike Topography for Bone Tissue Engineering**	ACS applied material	10.1021/acsami.7b01176	
Yang, F.; Xu, C. Y.; Kot	**Characterization of neural stem cells on electrospun poly(L-lactic acid) nanofibrous scaffold**	Journal of biomateria	10.1163/1568562042459733	Fiber topography not studied
Yi Xia; Shuang Li; Chu	**A multivalent polyanion-dispersed carbon nanotube toward highly bioactive nanostructured fibrous stem cell scaffolds**	Applied Materials To	10.1016/j.apmt.2019.07.006	CNT use does not allow for isolation of topographical effects
YongBok Kim; GeunH	**Highly roughened polycaprolactone surfaces using oxygen plasma-etching and in vitro mineralization for bone tissue regeneration: Fabrication, characterization, and cellular activities**	Colloids and Surface	10.1016/j.colsurfb.2014.11.033	Coating with SBF doe not allow for isolation of topographical effects
Yu, T.; Gleeson, S. E.; I	**Electrospun poly(epsilon-caprolactone) nanofiber shish kebabs mimic mineralized bony surface features**	Journal of Biomedica	10.1002/jbm.b.34207	
Yu, T.; Marcolongo, M	**Electrospun Poly(ε-caprolactone) nanofiber shish kebabs mimicking mineralized collagen fibrils in bone**	J. Orthop. Res.		Article could not be found
Yu Luo; Shige Wang;	**Carbon nanotube-incorporated multilayered cellulose acetate nanofibers for tissue engineering applications**	Carbohydrate Polym	10.1016/j.carbpol.2012.08.069	CNT use does not allow for isolation of topographical effects
Zamani, F.; Amani-Tel	**The influence of surface nanoroughness of electrospun PLGA nanofibrous scaffold on nerve cell adhesion and proliferation**	J. Mater. Sci. Mater. I	10.1007/s10856-013-4905-6	
Zandén, C.; Hellström	**Stem cell responses to plasma surface modified electrospun polyurethane scaffolds**	Nanomed. Nanotech	10.1016/j.nano.2014.01.010	
Zhang, C. H.; Cheng, I	**Nanoporous structured carbon nanofiber-bioactive glass composites for skeletal tissue regeneration**	Journal of Materials	10.1039/c5tb00921a	CNT use does not allow for isolation of topographical effects
Zhi Wang; Quan Qing	**Effects of scaffold surface morphology on cell adhesion and survival rate in vitreous cryopreservation of tenocyte-scaffold constructs**	Applied Surface Scie	10.1016/j.apsusc.2016.01.187	No electrospinning involved
Zhou, Q. H.; Xie, J.; Ba	**Engineering aligned electrospun PLLA microfibers with nano-porous surface nanotopography for modulating the responses of vascular smooth muscle cells**	Journal of Materials	10.1039/c5tb00051c	
