# Supplementary material for: How Fiber Surface Topography Affects Interactions between Cells and Electrospun Scaffolds: A Systematic Review
Source: Polymers (Basel). 2022 Jan 5;14(1):209. doi: 10.3390/polym14010209 (PMC8747153; doi:10.3390/polym14010209)
Supplement: Supplementary file 1 [file polymers-14-00209-s001.zip › Supplementary Information File S2.pdf]

Data to be extracted	Data
Title	
Author(s)	
DOI	
Year of publication	
Study objective as stated	
Electrospun Material	
Fiber surface topography modification method	
Other modifications that arose from the topography modification (e.g. chemical modification)	
Other modifications performed independently from topography modification that may have a bearing on the results	
Fiber surface topography characterization performed, if any	
Results of surface topography characterization, if any	
Control/comparator used for cell assay	
Cells used for study	
Cell viability outcome, if any	

**Cell adhesion outcome, if any**	
**Cell proliferation outcome, if any**	
**Cell differentiation outcome, if any**	
**Other cell behavior data**	
**Author conclusions regarding effect of surface topography on cells**	
