# Supplementary material for: How Fiber Surface Topography Affects Interactions between Cells and Electrospun Scaffolds: A Systematic Review
Source: Polymers (Basel). 2022 Jan 5;14(1):209. doi: 10.3390/polym14010209 (PMC8747153; doi:10.3390/polym14010209)
Supplement: Supplementary file 1 [file polymers-14-00209-s001.zip › Supplementary Information File S3.pdf]

Data to be extracted	Data
**Title**	Tailoring surface nanoroughness of electrospun scaffolds for skeletal tissue engineering
**Author(s)**	Honglin Chen; Xiaobin Huang; Minmin Zhang; Febriyani Damanik; Matthew B. Baker; Anne Leferink; Huipin Yuan; Roman Truckenmüller
**DOI**	10.1016/j.actbio.2017.07.003
**Year of publication**	2017
**Study objective as stated**	Investigating the influence of electrospun fiber surface nanoroughness on the skeletal differentiation of hMSCs.
**Electrospun Material**	Poly (ethylene oxide terephthalate)/poly (butylene terephthalate) (PEOT/PBT, weight ratio of PEOT/PBT=55/45)
**Fiber surface topography modification method**	Relative humidity is varied for the electrospinning process between 20%, 50%, and 70%, topography modified by phase separation (vapor-induced and thermal-induced).
**Other modifications that arose from the topography modification (e.g. chemical modification)**	Higher humidity (higher roughness) led to more hydrophobic scaffolds.
**Other modifications performed independently from topography modification that may have a bearing on the results**	For differentiation, cell seeded scaffolds were placed in Basic Medium and Osteogenic/Chondrogenic Medium and compared.
**Fiber surface topography characterization performed, if any**	SEM, AFM
**Results of surface topography characterization, if any**	SEM: the images show a significant difference in surface topography, with higher nanoporosity for higher relative humidity. AFM: significant increases in R_a, pore depth, and surface area for each increase in humidity.
**Control/comparator used for cell assay**	The different scaffolds (20,50 and 70%) were compared to each other.
**Cells used for study**	Human Mesenchymal Stem Cells (hMSCs)
**Cell viability outcome, if any**	No significant differences in metabolic activity

**Cell adhesion outcome, if any**	
**Cell proliferation outcome, if any**	
**Cell differentiation outcome, if any**	ALP: Increased osteogenic differentiation with higher roughness at day 21. GAG: No significant differences in chondrogenic differentiation. Gene expression: different osteogenic improvements for each roughness. Lower roughness yields better chondrogenic differentiation.
**Other cell behavior data**	
**Author conclusions regarding effect of surface topography on cells**	Our work opens new avenues for tailoring surface nanoroughness of electrospun scaffolds to control cellular response of hMSCs

Data to be extracted	Data
**Title**	Superhydrophilic poly(L-lactic acid) electrospun membranes for biomedical applications obtained by argon and oxygen plasma treatment
**Author(s)**	Correia, D. M.; Ribeiro, C.; Botelho, G.; Borges, J.; Lopes, C.; Vaz, F.; Carabineiro, S. A. C.; Machado, A. V.; Lanceros-Mendez, S.
**DOI**	10.1016/j.apsusc.2016.02.121
**Year of publication**	2016
**Study objective as stated**	The effects of O2 and Ar plasma treatments on the surface wettability of PLLA electrospun membranes and films is investigated. Furthermore, the influence of plasma treatment on fiber morphology, surface composition and degree of crystallinity is also evaluated.
**Electrospun Material**	Poly(L-lactic acid) (PLLA)
**Fiber surface topography modification method**	O2 and Ar Plasma treatment
**Other modifications that arose from the topography modification (e.g. chemical modification)**	Carbon decrease, oxygen increase. Formation of polar groups leads to improved hydrophilicity.
**Other modifications performed independently from topography modification that may have a bearing on the results**	
**Fiber surface topography characterization performed, if any**	SEM
**Results of surface topography characterization, if any**	O2 plasma application results in flat fibers.
**Control/comparator used for cell assay**	Negative and positive controls, non-plasma-treated fibers.<br>The same assay was performed on plasma treated and untreated PLLA films.
**Cells used for study**	MC3T3-E1 pre-osteoblast cells

**Cell viability outcome, if any**	No significant differences with respect to control
**Cell adhesion outcome, if any**	
**Cell proliferation outcome, if any**	
**Cell differentiation outcome, if any**	
**Other cell behavior data**	
**Author conclusions regarding effect of surface topography on cells**	The obtained samples are not cytotoxic.

Data to be extracted	Data
**Title**	Surface modification of electrospun PVA/chitosan nanofibers by dielectric barrier discharge plasma at atmospheric pressure and studies of their mechanical properties and biocompatibility
**Author(s)**	Punamshree Das; Namita Ojah; Raghuram Kandimalla; Kiranjyoti Mohan; Dolly Gogoi; Swapan Kumar Dolui; Arup Jyoti Choudhury
**DOI**	10.1016/j.ijbiomac.2018.03.115
**Year of publication**	2018
**Study objective as stated**	surface modification of PVA/Cs nanofibers by low temperature plasma treatment and subsequent studies of their physico-chemical and mechanical properties and biocompatibility to explore possible biomedical applications
**Electrospun Material**	poly(vinyl alcohol) (PVA) and Chitosan (Cs) combined into PVA-Cs
**Fiber surface topography modification method**	dielectric barrier discharge (DBD) O_2 and Ar plasma at atmospheric pressure
**Other modifications that arose from the topography modification (e.g. chemical modification)**	Chemical modifications
**Other modifications performed independently from topography modification that may have a bearing on the results**	
**Fiber surface topography characterization performed, if any**	FESEM, AFM
**Results of surface topography characterization, if any**	AFM shows increased nanoscale roughness on fibers, especially with O_2 plasma.
**Control/comparator used for cell assay**	
**Cells used for study**	Mouse Fibroblasts
**Cell viability outcome, if any**	Cytocompatibility increased, especially with O2 plasma.

**Cell adhesion outcome, if any**	
**Cell proliferation outcome, if any**	Cell viability decreases over time, so cell proliferation is negatively affected.<br>No statistical data is made available to this end.
**Cell differentiation outcome, if any**	
**Other cell behavior data**	
**Author conclusions regarding effect of surface topography on cells**	Both PVA/Cs/Ar and PVA/Cs/O2 nanofibers are observed to exhibit better cytocompatibility than AASF

Data to be extracted	Data
**Title**	Preparation and characterization of an electrospun polycaprolactone (PCL) fibrous mat and multi-layered PCL scaffolds having a nanosized pattern-surface for tissue regeneration.
**Author(s)**	Jeon, H.; Kim, G.
**DOI**	10.1039/c3tb21230k
**Year of publication**	2014
**Study objective as stated**	Demonstrate the feasibility of the physically roughened surface of the electrospun fibers as a tissue regenerative material
**Electrospun Material**	PCL
**Fiber surface topography modification method**	O2 low-frequency plasma treatment
**Other modifications that arose from the topography modification (e.g. chemical modification)**	Slight increase in oxygen, decrease in carbon content.
**Other modifications performed independently from topography modification that may have a bearing on the results**	
**Fiber surface topography characterization performed, if any**	SEM, surface roughness tester (interferometry)
**Results of surface topography characterization, if any**	Markedly increased roughness after plasma treatment
**Control/comparator used for cell assay**	Untreated PCL electrospun scaffold
**Cells used for study**	MG63 (osteosarcoma cells)
**Cell viability outcome, if any**	

**Cell adhesion outcome, if any**	Increased initial cell adhesion in plasma-treated scaffolds.
**Cell proliferation outcome, if any**	Significantly more proliferation on plasma-treated scaffold, based on nucleus count and ALP activity.
**Cell differentiation outcome, if any**	
**Other cell behavior data**	
**Author conclusions regarding effect of surface topography on cells**	The treated PCL mats exhibited outstanding initial cell attachment and proliferation, ALP activity, and calcium deposition

Data to be extracted	Data
**Title**	Fabrication of polycaprolactone electrospun fibers with different hierarchical structures mimicking collagen fibrils for tissue engineering scaffolds
**Author(s)**	Lin Jiang; Liwei Wang; Nathan Wang; Shaoqin Gong; Lixia Wang; Qian Li; Changyu Shen; Lih-Sheng Turng
**DOI**	10.1016/j.apsusc.2017.08.005
**Year of publication**	2018
**Study objective as stated**	3T3 mouse fibroblast cells were first used for preliminary cytocompatibility tests. Human umbilical vein endothelial cells (HUVECs) were then cultured to further investigate the endothelial cell response on smooth-surface PCL fibers, nanoporous PCL fibers, and nano-protruding shish–kebab-structured fibers
**Electrospun Material**	PCL
**Fiber surface topography modification method**	Vapor induced phase separation for nanopores, Shish-kebab structures by self-cristallization
**Other modifications that arose from the topography modification (e.g. chemical modification)**	Different fiber diameter
**Other modifications performed independently from topography modification that may have a bearing on the results**	
**Fiber surface topography characterization performed, if any**	SEM, AFM
**Results of surface topography characterization, if any**	The shish-kebab fibers show a larger height gradient in their surface features
**Control/comparator used for cell assay**	Solid PCL fibers
**Cells used for study**	Swiss mouse NIH 3T3 fibroblast cells (cytocompatibility)<br>Human umbilical vein endothelial cells (HUVEC) (viability, proliferation, adhesion)

**Cell viability outcome, if any**	3T3s: higher cell viability for structured scaffolds as opposed to solid.<br>HUVECs: best results for the nanoporous scaffolds
**Cell adhesion outcome, if any**	
**Cell proliferation outcome, if any**	3T3s: higher cell proliferation for structured scaffolds as opposed to solid<br>HUVECs: best results for the nanoporous scaffolds
**Cell differentiation outcome, if any**	
**Other cell behavior data**	Cells on nanoporous scaffolds had better cell-cell interactions
**Author conclusions regarding effect of surface topography on cells**	The 3T3 fibroblast cell and HUVEC cell assays on the electrospun PCL fibrous scaffolds with different hierarchical structures showed that cell viability and proliferation improved when the fibers were decorated by hierarchical structures

Data to be extracted	Data
**Title**	Fabrication of shish–kebab structured poly(ε-caprolactone) electrospun nanofibers that mimic collagen fibrils: Effect of solvents and matrigel functionalization
**Author(s)**	Xin Jing; Hao-Yang Mi; Travis M. Cordie; Max R. Salick; Xiang-Fang Peng; Lih-Sheng Turng
**DOI**	10.1016/j.polymer.2014.08.061
**Year of publication**	2014
**Study objective as stated**	We investigated the effect of different solvents on the formation of PCL shish-kebab structures on the electrospun PCL nanofibrous scaffolds to develop nanotopography that mimic the structure of native collagen fibrils. Then the matrigel was covalently immobilized on the shish-kebab structured PCL nanofibrous scaffolds to better mimic the collagen fibrils not only from the aspects of structure and surface features but also for biocompatibility enhancement.
**Electrospun Material**	PCL
**Fiber surface topography modification method**	Shish kebab induction via incubation in acetic acid or pentyl acetate
**Other modifications that arose from the topography modification (e.g. chemical modification)**	Hydrophobicity, small chemical variations detected by FTIR
**Other modifications performed independently from topography modification that may have a bearing on the results**	
**Fiber surface topography characterization performed, if any**	SEM
**Results of surface topography characterization, if any**	Fiber diameter. Visualization of SKs shows that the process was effective at creating Time-dependently growing SKs.
**Control/comparator used for cell assay**	Smooth PCL scaffold
**Cells used for study**	HEF1 fibroblast cells

**Cell viability outcome, if any**	No significant differences
**Cell adhesion outcome, if any**	
**Cell proliferation outcome, if any**	Significantly increased proliferation after 10 and 15 days
**Cell differentiation outcome, if any**	
**Other cell behavior data**	
**Author conclusions regarding effect of surface topography on cells**	The HEF1 fibroblast cell assays on different nanofibrous scaffolds with and Without shish-kebab structure showed that PCL nanofibers with kebabs on the surface enhanced cell adhesion and proliferation due to the difference in topography of the scaffolds.

Data to be extracted	Data
**Title**	Fabrication of fluffy shish-kebab structured nanofibers by electrospinning, CO2 escaping foaming and controlled crystallization for biomimetic tissue engineering scaffolds
**Author(s)**	Xin Jing; Heng Li; Hao-Yang Mi; Yue-Jun Liu; Yi-Min Tan
**DOI**	10.1016/j.cej.2019.04.194
**Year of publication**	2019
**Study objective as stated**	we focused on the biocompatibility of the 3D fluffy nanofibrous scaffolds, and investigated their potential application as tissue engineering scaffolds for 3D tissue regeneration
**Electrospun Material**	PCL
**Fiber surface topography modification method**	Formation of SKs through incubation in Pentyl acetate/PCL solution
**Other modifications that arose from the topography modification (e.g. chemical modification)**	
**Other modifications performed independently from topography modification that may have a bearing on the results**	
**Fiber surface topography characterization performed, if any**	SEM
**Results of surface topography characterization, if any**	SKs visible on treated samples
**Control/comparator used for cell assay**	2d and 3D PCL scaffolds
**Cells used for study**	NIH 3T3 mouse fibroblasts and human fibroblasts
**Cell viability outcome, if any**	No significant difference

**Cell adhesion outcome, if any**	
**Cell proliferation outcome, if any**	Significantly more proliferation on 3d sk pcl than on 3d pcl
**Cell differentiation outcome, if any**	
**Other cell behavior data**	
**Author conclusions regarding effect of surface topography on cells**	The prepared 3D scaffold was more favorable for human fibroblast cell growth, with enhanced cell attachment, higher cell viability and better cell infiltration behavior.

Data to be extracted	Data
**Title**	Effect of electrospun poly(D,L-lactide) fibrous scaffold with nanoporous surface on attachment of porcine esophageal epithelial cells and protein adsorption
**Author(s)**	Leong, Meng Fatt; Chian, Kerm Sin; Mhaisalkar, Priyadarshini S.; Ong, Wey Feng; Ratner, Buddy D.
**DOI**	10.1002/jbm.a.32061
**Year of publication**	2009
**Study objective as stated**	The effects of electrospun fiber nano-porosity on cell attachment and protein adsorption were studied. We hypothesize that the creation of nanoporous fiber surface influences the initial cell attachment
**Electrospun Material**	poly(D,L-lactide) (PLA)
**Fiber surface topography modification method**	Use of a non-water-soluble solvent in order to cause vapor induced phase separation and therewith nanoporosity.
**Other modifications that arose from the topography modification (e.g. chemical modification)**	
**Other modifications performed independently from topography modification that may have a bearing on the results**	
**Fiber surface topography characterization performed, if any**	SEM
**Results of surface topography characterization, if any**	Qualitative appreciation of nanoporosity on the modified fiber's surface
**Control/comparator used for cell assay**	Solid electrospun fiber scaffold
**Cells used for study**	Porcine esophageal epithelial cells (PEECs)
**Cell viability outcome, if any**	

**Cell adhesion outcome, if any**	Improved cell adhesion on nanoporous fiber scaffolds as opposed to solid fiber.
**Cell proliferation outcome, if any**	
**Cell differentiation outcome, if any**	
**Other cell behavior data**	
**Author conclusions regarding effect of surface topography on cells**	it has been shown that the fibrous nature of the electrospun scaffolds promote cell attachment as compared with the solvent-cast film, and the nanoporous fiber scaffolds further enhance this interaction.

Data to be extracted	Data
**Title**	Ultraporous interweaving electrospun microfibers from PCL–PEO binary blends and their inflammatory responses
**Author(s)**	Li, Yan-Fang; Rubert, Marina; Aslan, Hüsnü; Yu, Ying; Howard, Kenneth A.; Dong, Mingdong; Besenbacher, Flemming; Chen, Menglin
**DOI**	10.1039/c3nr06197c
**Year of publication**	2014
**Study objective as stated**	Based on the morphology and structure characterization, the formation mechanism of the novel structure could be proposed
**Electrospun Material**	PCL / PEO (pure/mixed)
**Fiber surface topography modification method**	Variation in PCL/PEO ratio
**Other modifications that arose from the topography modification (e.g. chemical modification)**	Different material i.e. different chemical and mechanical properties
**Other modifications performed independently from topography modification that may have a bearing on the results**	
**Fiber surface topography characterization performed, if any**	SEM
**Results of surface topography characterization, if any**	Qualitative assessment of the structural differences induced by increasing the PEO ratio.
**Control/comparator used for cell assay**	Pure solid PCL scaffolds
**Cells used for study**	RAW 264.7 cells
**Cell viability outcome, if any**	No statement is made about significance of results, but the cell viability appears no to differ much among evaluated scaffolds.

**Cell adhesion outcome, if any**	Adhesion was not assessed quantitatively, but all evaluated scaffolds allowed for adhesion.
**Cell proliferation outcome, if any**	
**Cell differentiation outcome, if any**	
**Other cell behavior data**	
**Author conclusions regarding effect of surface topography on cells**	The blended fibers with distinguished morphologies were found to be biocompatible

Data to be extracted	Data
**Title**	Biomimetic Nanocomposites to Control Osteogenic Differentiation of Human Mesenchymal Stem Cells
**Author(s)**	Liao, Susan; Nguyen, Luong T. H.; Ngiam, Michelle; Wang, Charlene; Cheng, Ziyuan; Chan, Casey K.; Ramakrishna, Seeram
**DOI**	10.1002/adhm.201300207
**Year of publication**	2014
**Study objective as stated**	we have investigated the specific micro-environmental cues that are necessary to induce osteogenic differentiation of MSCs
**Electrospun Material**	poly(L -lactic acid) (PLLA)
**Fiber surface topography modification method**	Phase separation with DCM solvent (no further explanation)
**Other modifications that arose from the topography modification (e.g. chemical modification)**	Significantly different fiber diameter
**Other modifications performed independently from topography modification that may have a bearing on the results**	
**Fiber surface topography characterization performed, if any**	SEM
**Results of surface topography characterization, if any**	Qualitative assessment: visible nanopores on the fibers.
**Control/comparator used for cell assay**	Solid nanofibrous PLLA scaffold
**Cells used for study**	Human Mesenchymal Stem Cells (hMSCs)
**Cell viability outcome, if any**	No significant difference is indicated

**Cell adhesion outcome, if any**	
**Cell proliferation outcome, if any**	No significant difference is indicated
**Cell differentiation outcome, if any**	No significant difference is indicated
**Other cell behavior data**	
**Author conclusions regarding effect of surface topography on cells**	

Data to be extracted	Data
**Title**	Controlling the surface structure of electrospun fibers: Effect on endothelial cells and blood coagulation
**Author(s)**	Mertgen, A. S.; Yazgan, G.; Guex, A. G.; Fortunato, G.; Muller, E.; Huber, L.; Schneider, R.; Brunelli, M.; Rossi, R. M.; Maniura-Weber, K.; Rottmar, M.
**DOI**	10.1116/1.5047668
**Year of publication**	2018
**Study objective as stated**	the behavior of HUVECs as well as platelet activation and blood coagulation on electrospun membranes with distinct surface structures was studied.
**Electrospun Material**	PCL
**Fiber surface topography modification method**	Different solvents (VIPS)
**Other modifications that arose from the topography modification (e.g. chemical modification)**	
**Other modifications performed independently from topography modification that may have a bearing on the results**	
**Fiber surface topography characterization performed, if any**	SEM
**Results of surface topography characterization, if any**	SEM images show a very different fiber surface structure.
**Control/comparator used for cell assay**	Non-porous (but grooved) microfibers
**Cells used for study**	HUVECs
**Cell viability outcome, if any**	

**Cell adhesion outcome, if any**	
**Cell proliferation outcome, if any**	Proliferation was significantly increased in porous scaffolds
**Cell differentiation outcome, if any**	
**Other cell behavior data**	
**Author conclusions regarding effect of surface topography on cells**	results provided in this study demonstrate comparable HUVEC behavior on MF and structured MF membranes

Data to be extracted	Data
**Title**	Fiber diameter and texture of electrospun PEOT/PBT scaffolds influence human mesenchymal stem cell proliferation and morphology, and the release of incorporated compounds
**Author(s)**	Lorenzo Moroni; Ruud Licht; Jan de Boer; Joost R. de Wijn; Clemens A. van Blitterswijk
**DOI**	10.1016/j.biomaterials.2006.05.027
**Year of publication**	2006
**Study objective as stated**	The aim of this study was to characterize electrospun scaffolds of a PEOT/PBT copolymer in order to assess their possible use as constructs for tissue engineering and controlled drug release applications.
**Electrospun Material**	copolymers of poly(ethyleneoxide terephthalate)–poly(butylene terephthalate) (PEOT/PBT)
**Fiber surface topography modification method**	Solvent variation (VIPS).
**Other modifications that arose from the topography modification (e.g. chemical modification)**	Changes in general scaffold morphology.
**Other modifications performed independently from topography modification that may have a bearing on the results**	
**Fiber surface topography characterization performed, if any**	SEM
**Results of surface topography characterization, if any**	Different solvents, as well as different solvent ratios, produced very different fiber surface structures
**Control/comparator used for cell assay**	Smooth surface scaffolds
**Cells used for study**	Human mesenchymal stem cells(hMSCs)
**Cell viability outcome, if any**	

**Cell adhesion outcome, if any**	
**Cell proliferation outcome, if any**	Higher cell proliferation on nanoporous scaffold compared to smooth scaffold
**Cell differentiation outcome, if any**	
**Other cell behavior data**	
**Author conclusions regarding effect of surface topography on cells**	If nanoporous fibers are considered, a significantly higher cell activity was found with respect to non-porous fibers with the same diameter

Data to be extracted	Data
**Title**	Surface modification of electrospun fibre meshes by oxygen plasma for bone regeneration
**Author(s)**	Nandakumar, A.; Birgani, Z. T.; Santos, D.; Mentink, A.; Auffermann, N.; van der Werf, K.; Bennink, M.; Moroni, L.; van Blitterswijk, C.; Habibovic, P.
**DOI**	10.1088/1758-5082/5/1/015006
**Year of publication**	2013
**Study objective as stated**	The aim of this study was to evaluate protein adsorption, metabolic activity, proliferation and osteogenic differentiation of human mesenchymal stromal cells (hMSCs) on oxygenplasma treated electrospun PolyActive^{TM}(PA) meshes
**Electrospun Material**	PolyActive^{TM}(PA) , which is a family of block copolymers of poly (ethylene oxide terephthalate) and poly (butyleneterephthalate) (PEOT/PBT)
**Fiber surface topography modification method**	O2 Plasma
**Other modifications that arose from the topography modification (e.g. chemical modification)**	The oxygen/carbon ratio increased after treatment.
**Other modifications performed independently from topography modification that may have a bearing on the results**	
**Fiber surface topography characterization performed, if any**	SEM, AFM
**Results of surface topography characterization, if any**	Surface roughness increased exponentially with treatment time, significantly after 15 min. SEM images show roughness increasing with treatment time.
**Control/comparator used for cell assay**	Untreated fibers
**Cells used for study**	hMSCs
**Cell viability outcome, if any**	

**Cell adhesion outcome, if any**	Adhesion appeared to be increased (qualitative)
**Cell proliferation outcome, if any**	No significant differences in ALP, DNA, or metabolic activity
**Cell differentiation outcome, if any**	Some osteogenic markers (BSP, ON) were significantly increased with treatment time. One was decreased with 15 min treatment but was equal in 30 min-treated and untreated fibers. Other osteogenic markers were not significantly altered.
**Other cell behavior data**	
**Author conclusions regarding effect of surface topography on cells**	Cell metabolic activity in different scaffold groups was similar up to 7 days of culture and so was the amount of DNA on day 7. The effects on the expression of BSP and ON were solely the result of surface modification.

Data to be extracted	Data
**Title**	Effects of nozzle type atmospheric dry air plasma on L929 fibroblast cells hybrid poly (ε-caprolactone)/chitosan/poly (ε-caprolactone) scaffolds interactions
**Author(s)**	Ozan Ozkan; Hilal Turkoglu Sasmazel
**DOI**	10.1016/j.jbiosc.2016.01.004
**Year of publication**	2016
**Study objective as stated**	using nozzle type atmospheric dry air plasma modification, it was aimed to introduce/enhance reactive functional groups and/or topographical alterations to the surface in order to improve cell-material interactions
**Electrospun Material**	PCL/chitosan
**Fiber surface topography modification method**	Air Plasma
**Other modifications that arose from the topography modification (e.g. chemical modification)**	Decrease in carbon, increase in oxygen, significant increase in fiber diameter
**Other modifications performed independently from topography modification that may have a bearing on the results**	
**Fiber surface topography characterization performed, if any**	SEM, AFM
**Results of surface topography characterization, if any**	Increase in fiber surface roughness after plasma modification
**Control/comparator used for cell assay**	TCPS Control, Unmodified scaffold
**Cells used for study**	Mouse fibroblasts
**Cell viability outcome, if any**	Increased (statistical significance unclear)

**Cell adhesion outcome, if any**	
**Cell proliferation outcome, if any**	Clearly increased
**Cell differentiation outcome, if any**	
**Other cell behavior data**	
**Author conclusions regarding effect of surface topography on cells**	Nozzle type atmospheric dry air plasma modification was found to be a useful tool in enhancing both the initial cell-material interactions and the performances of the scaffolds over a long period

Data to be extracted	Data
Title	Engineered nanotopography on electrospun PLLA microfibers modifies RAW 264.7 cell response
Author(s)	Schaub, N. J.; Britton, T.; Rajachar, R.; Gilbert, R. J.
DOI	10.1021/am402827g
Year of publication	2013
Study objective as stated	One goal of this study was to develop a new procedure to place defined nanotopography onto the surface of electrospun fibers. To determine if the presence of nanoscale depressions altered cell behavior, RAW 264.7 cells were cultured on fibers with and without nanoscale depressions
Electrospun Material	Poly-L-lactic Acid (PLLA)
Fiber surface topography modification method	Non-solvent induced Phase separation
Other modifications that arose from the topography modification (e.g. chemical modification)	
Other modifications performed independently from topography modification that may have a bearing on the results	
Fiber surface topography characterization performed, if any	SEM
Results of surface topography characterization, if any	Different concentrations of nonsolvent caused different nanoscale depressions to appear in a nonlinear fashion.
Control/comparator used for cell assay	Porous and smooth fiber scaffolds of similar and different fiber diameters were compared
Cells used for study	RAW 264.7 Macrophages

**Cell viability outcome, if any**	No significant differences
**Cell adhesion outcome, if any**	No significant differences
**Cell proliferation outcome, if any**	
**Cell differentiation outcome, if any**	
**Other cell behavior data**	
**Author conclusions regarding effect of surface topography on cells**	Although there was a change in cell morphology on fibers with nanotopography, there was no change in cell metabolism or the number of adherent cells.

Data to be extracted	Data
Title	Atmospheric plasma surface modifications of electrospun PCL/chitosan/PCL hybrid scaffolds by nozzle type plasma jets for usage of cell cultivation
Author(s)	Seda Surucu; Kai Masur; Hilal Turkoglu Sasmazel; Thomas Von Woedtke; Klaus Dieter Weltmann
DOI	10.1016/j.apsusc.2016.05.123
Year of publication	2016
Study objective as stated	The aim of this study was to achieve better cell adhesion and growth efficiency of PCL/chitosan/PCL electrospun layer by layer hybrid scaffolds
Electrospun Material	Poly(epsilon-caprolactone)(PCL) and Chitosan (Cs) -> PCL-Cs
Fiber surface topography modification method	Ar Plasma, Air plasma
Other modifications that arose from the topography modification (e.g. chemical modification)	Decrease in C, increase in O
Other modifications performed independently from topography modification that may have a bearing on the results	
Fiber surface topography characterization performed, if any	SEM
Results of surface topography characterization, if any	Ar Plasma made fiber surfaces rougher, Air plasma made fibers flat.
Control/comparator used for cell assay	Untreated scaffolds, TCPS Petri dishes
Cells used for study	MRC5 human fibroblasts
Cell viability outcome, if any	Improved viability (Air plasma)

**Cell adhesion outcome, if any**	
**Cell proliferation outcome, if any**	Improved proliferation on both air plasma and Ar Plasma
**Cell differentiation outcome, if any**	
**Other cell behavior data**	
**Author conclusions regarding effect of surface topography on cells**	Atmospheric surface plasma treatments were effective on the changes of the Nanotopography and/or functionality of the scaffolds, resulting with enhance Fibroblast cell attachment and proliferation

Data to be extracted	Data
**Title**	Nanotopography featured polycaprolactone/polyethyleneoxide microfibers modulate endothelial cell response
**Author(s)**	Taskin, Mehmet Berat; Xia, Dan; Besenbacher, Flemming; Dong, Mingdong; Chen, Menglin
**DOI**	10.1039/C7NR03326E
**Year of publication**	2017
**Study objective as stated**	create 3D nanotopography featured substrates for HUVEC interactions in terms of focal adhesions, cell spreading and proliferation
**Electrospun Material**	PCL/PEO
**Fiber surface topography modification method**	Varying PCL:PEO ratios with a solvent/non-solvent mixture.
**Other modifications that arose from the topography modification (e.g. chemical modification)**	Material properties varied according to the properties of PCL and PEO, as well As the fiber diameter increasing significantly from mesh I to mesh VII. Hydrophobicity was reduced, and macroscale surface area increased.
**Other modifications performed independently from topography modification that may have a bearing on the results**	
**Fiber surface topography characterization performed, if any**	SEM, AFM
**Results of surface topography characterization, if any**	Mesh I had secondary polymeric lamellae with about 10nm distance, whereas Mesh VII showed a shish-kebab structure with the same lamellae superimposed on it. SEM showed longitudinal grooves in Mesh I.
**Control/comparator used for cell assay**	Pure PCL Scaffold
**Cells used for study**	HUVECs
**Cell viability outcome, if any**	

**Cell adhesion outcome, if any**	Mesh I showed a significant increase in both measured focal adhesion markers. Mesh VII showed a significant increase in one of them.
**Cell proliferation outcome, if any**	Cells proliferated significantly faster on Mesh I than any other scaffold. They Proliferated slower on Mesh VII than any other sample.
**Cell differentiation outcome, if any**	
**Other cell behavior data**	
**Author conclusions regarding effect of surface topography on cells**	Furthermore, nascent adhesion complex on such 3D nanotopography featured Substrates was evidenced by pFAK and vinculin expression, even on the PEO dominant substrate. The nanotopography featured PCL-PEO fibers enhanced HUVEC proliferation rate compared to their smooth film counterparts

Data to be extracted	Data
**Title**	Nanoporous fibers of type-I collagen coated poly(l -lactic acid) for enhancing primary hepatocyte growth and function
**Author(s)**	Wang, Ting; Feng, Zhang-Qi; Leach, Michelle K.; Wu, Jinghang; Jiang, Qing
**DOI**	10.1039/C2TB00195K
**Year of publication**	2013
**Study objective as stated**	to combine the advantages of fibrous and porous nanotopographies together to obtain better cellular activity and liver-specific function of primary hepatocytes than that provided by fibrous topography alone
**Electrospun Material**	PLLA coated with Type-I Collagen
**Fiber surface topography modification method**	Non-solvent induced phase separation
**Other modifications that arose from the topography modification (e.g. chemical modification)**	
**Other modifications performed independently from topography modification that may have a bearing on the results**	NH_3 Plasma treatment, collagen-I coating
**Fiber surface topography characterization performed, if any**	SEM
**Results of surface topography characterization, if any**	Intrafiber pores clearly visible
**Control/comparator used for cell assay**	Smooth fibers
**Cells used for study**	Primary rat hepatocytes
**Cell viability outcome, if any**	

**Cell adhesion outcome, if any**	Initial adhesion was not different in a statistically significant manner.
**Cell proliferation outcome, if any**	Cell proliferation was marked by a decrease in cell content in both the porous and Smooth sample, but the smooth sample had a significantly faster reduction in Cell count.
**Cell differentiation outcome, if any**	
**Other cell behavior data**	
**Author conclusions regarding effect of surface topography on cells**	These data indicate that the nanopores of the nPFs promoted cell adhesion, and have an obvious effect on the improvement of cell retention of primary hepatocytes over a long term culture period in vitro.

Data to be extracted	Data
**Title**	Polylactic Acid Nanofiber Scaffold Decorated with Chitosan Islandlike Topography for Bone Tissue Engineering
**Author(s)**	Xu, Ting; Yang, Hongyang; Yang, Dongzhi; Yu, Zhong-Zhen
**DOI**	10.1021/acsami.7b01176
**Year of publication**	2017
**Study objective as stated**	The objective of this research was to design nanosized topography, and the high bioactivity of chitosan onto PLA electrospun fibers surface to improve the cell biocompatibility of PLA fibrous membrane
**Electrospun Material**	PLA/Cs
**Fiber surface topography modification method**	Electrospinning at different temperatures
**Other modifications that arose from the topography modification (e.g. chemical modification)**	Different distribution of chitosan on the fiber
**Other modifications performed independently from topography modification that may have a bearing on the results**	
**Fiber surface topography characterization performed, if any**	SEM, AFM, TEM
**Results of surface topography characterization, if any**	Island size ca. 200 nm, island height ca. 50 nm, island distance 400-800 nm<br>Higher temperatures led to less, smaller islands.
**Control/comparator used for cell assay**	Positive/negative controls, pure PLA, PLA/Cs core-shell
**Cells used for study**	MC3T3-E1 Mouse preosteoblastic Cells
**Cell viability outcome, if any**	PLA/Cs islandlike topographical sample proved significantly better after 2 days than The pure PLA.

**Cell adhesion outcome, if any**	
**Cell proliferation outcome, if any**	Proliferation visibly increased on islandlike PLA/Cs topography as compared to All other samples
**Cell differentiation outcome, if any**	Osteogenic differentiation improved on both PLA/Cs samples (with and without Topographical cues). At some points in time, osteogenic markers were significantly Heightened for the island structure, but mostly the difference was insignificant
**Other cell behavior data**	
**Author conclusions regarding effect of surface topography on cells**	The results showed that PLA scaffolds with CS island-nanotopology surfaces were more favorable for the spread of cells

Data to be extracted	Data
**Title**	Poly(ε-caprolactone) Nanofibers with a Self-Induced Nanohybrid Shish-Kebab Structure Mimicking Collagen Fibrils
**Author(s)**	Wang, Xiaofeng; Salick, Max R.; Wang, Xiaodong; Cordie, Travis; Han, Wenjuan; Peng, Yiyan; Li, Qian; Turng, Lih-Sheng
**DOI**	10.1021/bm400928b
**Year of publication**	2013
**Study objective as stated**	to create self-induced nanohybrid shish-kebab structures (SINSKs) on PCL nanofibers to mimic the nanotopography of collagen nanofibrils in the ECM
**Electrospun Material**	PCL (other materials were electrospun but only the PCL scaffold was used for the Cell assay)
**Fiber surface topography modification method**	Shish-Kebab self-induction via depositing drops of a PCL/acetic acid/deionized water Solution.
**Other modifications that arose from the topography modification (e.g. chemical modification)**	
**Other modifications performed independently from topography modification that may have a bearing on the results**	
**Fiber surface topography characterization performed, if any**	SEM
**Results of surface topography characterization, if any**	An increasing concentration of the applied solution provides larger and more Defined kebabs
**Control/comparator used for cell assay**	Smooth fibers
**Cells used for study**	Swiss mouse NIH 3T3 ECACC fibroblasts
**Cell viability outcome, if any**	The highest solution concentration sample showed significantly increased viability

**Cell adhesion outcome, if any**	
**Cell proliferation outcome, if any**	No significant changes on day 10.
**Cell differentiation outcome, if any**	
**Other cell behavior data**	
**Author conclusions regarding effect of surface topography on cells**	The cell assays on different nanofibers with and without SINSK structure showed that the presence of kebabs and their size affected the cell attachment and proliferation by changing the surface area to volume ratio of the PCL nanofibers

Data to be extracted	Data
**Title**	Electrospun poly(epsilon-caprolactone) nanofiber shish kebabs mimic mineralized bony surface features
**Author(s)**	Yu, T.; Gleeson, S. E.; Li, C. Y.; Marcolongo, M.
**DOI**	10.1002/jbm.b.34207
**Year of publication**	2019
**Study objective as stated**	we investigate and quantify the nanofiber alignment and kebab periodicity of polycaprolactone NFSK templates on MC3T3 E1 preosteoblast cells over time
**Electrospun Material**	PCL
**Fiber surface topography modification method**	SK induction by incubation in a PCL/Pentyl acetate solution
**Other modifications that arose from the topography modification (e.g. chemical modification)**	
**Other modifications performed independently from topography modification that may have a bearing on the results**	
**Fiber surface topography characterization performed, if any**	SEM
**Results of surface topography characterization, if any**	Visible SK formation increasing with incubation time
**Control/comparator used for cell assay**	Smooth fibers
**Cells used for study**	MC3T3 E1 osteoblast-like cells
**Cell viability outcome, if any**	

**Cell adhesion outcome, if any**	
**Cell proliferation outcome, if any**	Significantly improved proliferation at every timepoint measured for the SK-samples
**Cell differentiation outcome, if any**	Significantly improved ALP activity in most timepoints
**Other cell behavior data**	
**Author conclusions regarding effect of surface topography on cells**	Our results indicate that the addition of a nanoscale roughness enhances osteoblast function and activity. Tuned shish kebob periodicity and size controlled cellular response in this system with kebab periodicity on the order of collagen spacing resulting in the highest degree of alkaline phosphatase activity among the groups examined

Data to be extracted	Data
**Title**	Novel nanoscale topography on poly(propylene carbonate)/poly(ε-caprolactone) electrospun nanofibers modifies osteogenic capacity of ADCs
**Author(s)**	Wang, Y.; Deng, J.; Fan, R.; Tong, A.; Zhang, X.; Zhou, L.; Zheng, Y.; Xu, J.; Guo, G.
**DOI**	10.1039/c5ra15841a
**Year of publication**	2015
**Study objective as stated**	We hypothesized that the hybrid scaffolds could have better chemical–physical Properties than pure PPC or PCL electrospun membranes. Our further aim was to investigate the attachment, colonization, and osteogenic differentiation of ADCs isolated from subcutaneous adipose tissue of rats on PPC/PCL hybrid electrospun scaffolds
**Electrospun Material**	Polypropylene carbonate (PPC)/PCL
**Fiber surface topography modification method**	Variation of the PPC/PCL weight ratio
**Other modifications that arose from the topography modification (e.g. chemical modification)**	Material properties, mechanical, thermal and surface behavior changed
**Other modifications performed independently from topography modification that may have a bearing on the results**	
**Fiber surface topography characterization performed, if any**	SEM, TEM
**Results of surface topography characterization, if any**	Increased fiber porosity with the addition of PCL
**Control/comparator used for cell assay**	Pure PPC fibers
**Cells used for study**	Adipose tissue-derived stromal cells (ADCs)

**Cell viability outcome, if any**	Cell viability and metabolic activity significantly increased on PPC/PCL blend
**Cell adhesion outcome, if any**	
**Cell proliferation outcome, if any**	Cell proliferation only significantly different on day 4, whereas day 2 and day 6 show No statistically significant difference between samples.
**Cell differentiation outcome, if any**	Significantly better differentiation on PPC/PCL blend than pure PPC
**Other cell behavior data**	
**Author conclusions regarding effect of surface topography on cells**	ADCs cultured on electrospun fibers with inter-surface-connected pores show increased cellular adhesion, proliferation and differentiation than those cultured on the fiber with blind-holes

Data to be extracted	Data
**Title**	The influence of surface nanoroughness of electrospun PLGA nanofibrous scaffold on nerve cell adhesion and proliferation
**Author(s)**	Zamani, F.; Amani-Tehran, M.; Latifi, M.; Shokrgozar, M. A.
**DOI**	10.1007/s10856-013-4905-6
**Year of publication**	2013
**Study objective as stated**	It is hypothesized that the creation of different shapes of nanofibrous surfaces influences the initial cell attachment and the amount of protein that can be absorbed in the electrospun scaffold
**Electrospun Material**	PLGA (LA/GA 85/15)
**Fiber surface topography modification method**	Changing polymer weight ratio, solvent ratio and relative humidity (phase Separation)
**Other modifications that arose from the topography modification (e.g. chemical modification)**	
**Other modifications performed independently from topography modification that may have a bearing on the results**	
**Fiber surface topography characterization performed, if any**	SEM, AFM
**Results of surface topography characterization, if any**	Multiple surface roughness parameters were calculated with AFM. SEM showed Different roughnesses being achieved with a variation of the parameters, As well as porosity on the fibers
**Control/comparator used for cell assay**	TCPS Control, smooth scaffold
**Cells used for study**	A-172 Brain Glioblastoma
**Cell viability outcome, if any**	No significant differences

**Cell adhesion outcome, if any**	
**Cell proliferation outcome, if any**	Increased proliferation on rough scaffolds
**Cell differentiation outcome, if any**	
**Other cell behavior data**	
**Author conclusions regarding effect of surface topography on cells**	The results have demonstrated that the design of PLGA electrospun fibrous scaffolds composed of porous cylindrical fibres with nanometer roughness on their surfaces promotes the attachment, growth and proliferation of human nerve cells

Data to be extracted	Data
**Title**	Surface characterisation of oxygen plasma treated electrospun polyurethane fibres and their interaction with red blood cells
**Author(s)**	Carl Zandén; Marina Voinova; Julie Gold; Daniel Mörsdorf; Ingolf Bernhardt; Johan Liu
**DOI**	10.1016/j.eurpolymj.2012.01.004
**Year of publication**	2012
**Study objective as stated**	Oxygen plasma treatments were applied as a surface modification on electrospun PU and the effects on morphology, surface chemistry and wettability as a function of time were studied
**Electrospun Material**	PU
**Fiber surface topography modification method**	Oxygen plasma treatment with different time exposures
**Other modifications that arose from the topography modification (e.g. chemical modification)**	Increase of oxygen and nitrogen, decrease of carbon on the surface.
**Other modifications performed independently from topography modification that may have a bearing on the results, if any**	
**Fiber surface topography characterization performed, if any**	SEM
**Results of surface topography characterization, if any**	Qualitative evaluation: longer plasma application increased surface roughness
**Control/comparator used for cell assay**	Pristine PU electrospun scaffold was compared to 3 min treated plasma scaffold
**Cells used for study**	Red Blood cells
**Cell viability outcome, if any**	

**Cell adhesion outcome, if any**	Relatively low adhesion for both plasma-treated and pristine PU scaffolds.
**Cell proliferation outcome, if any**	
**Cell differentiation outcome, if any**	
**Other cell behavior data**	Cell morphology was affected: local protrusions were observed at cell-scaffold contact points.
**Author conclusions regarding effect of surface topography on cells**	The plasma-treated and pristine samples did not show large differences in cell behavior, although hydrophobicity was markedly different in both samples.

Data to be extracted	Data
**Title**	Stem cell responses to plasma surface modified electrospun polyurethane scaffolds
**Author(s)**	Zandén, C.; Hellström Erkenstam, N.; Padel, T.; Wittgenstein, J.; Liu, J.; Kuhn, H. G.
**DOI**	10.1016/j.nano.2014.01.010
**Year of publication**	2014
**Study objective as stated**	evaluate and compare how argon, oxygen and hydrogen plasma surface modifications on random and aligned electrospun polyurethane scaffolds affect the expansion, migration and differentiation of human embryonic stem cells as well as postnatal rodent neural stem cells
**Electrospun Material**	PU
**Fiber surface topography modification method**	Argon, Oxygen, Hydrogen Plasma
**Other modifications that arose from the topography modification (e.g. chemical modification)**	Changes in the O/C surface ratio
**Other modifications performed independently from topography modification that may have a bearing on the results**	For the differentiation assay, scaffolds were coated with laminin
**Fiber surface topography characterization performed, if any**	SEM
**Results of surface topography characterization, if any**	Reduced fiber diameter for the O-Plasma treatment. Other parameters did not Change much. Surface topography, however, was visibly affected but not Measured.
**Control/comparator used for cell assay**	Non-plasma treated fibers
**Cells used for study**	Human embryonic stem cells SA121<br>Rodent neural stem cells

**Cell viability outcome, if any**	
**Cell adhesion outcome, if any**	
**Cell proliferation outcome, if any**	Significant differences only on randomly oriented fibers for HeSC, particularly Ar. For all other Samples, no significant differences were found.
**Cell differentiation outcome, if any**	No significant differences
**Other cell behavior data**	
**Author conclusions regarding effect of surface topography on cells**	hESC attachment and proliferation were significantly improved through all three plasma treatments on both uncoated and protein coated scaffolds with random fiber orientation. In contrast, on aligned fibers the attachment levels were similar to those of smooth surfaces without modification, and no further increases from the plasma treatments were found. Directed orientation and migration of adult NSCs along fibers were observed on all samples. Differentiation of adult NSCs was found to be insensitive to roughness and surface chemistry of individual fibers

Data to be extracted	Data
**Title**	Engineering aligned electrospun PLLA microfibers with nano-porous surface nanotopography for modulating the responses of vascular smooth muscle cells
**Author(s)**	Zhou, Q. H.; Xie, J.; Bao, M.; Yuan, H. H.; Ye, Z. Y.; Lou, X. X.; Zhang, Y. Z.
**DOI**	10.1039/c5tb00051c
**Year of publication**	2015
**Study objective as stated**	We hypothesize that aligned electrospun ultrafine fibers endowed with nanotopographical features would also be able to present favorable physical cues, in a manner of correlating with the nanostructure geometry, to regulate the cellular responses of vSMCs in engineering vascular tissues
**Electrospun Material**	PLLA
**Fiber surface topography modification method**	Variation of humidity (VIPS)
**Other modifications that arose from the topography modification (e.g. chemical modification)**	
**Other modifications performed independently from topography modification that may have a bearing on the results**	
**Fiber surface topography characterization performed, if any**	SEM, AFM
**Results of surface topography characterization, if any**	Increased roughness with increasing relative humidity, going from 55 to 110 nm.
**Control/comparator used for cell assay**	Different relative humidities are compared among each other
**Cells used for study**	Vascular smooth muscle cells (vSMCs), in particular human arterial umbilical Smooth muscle cells

**Cell viability outcome, if any**	
**Cell adhesion outcome, if any**	Initial cell adhesion stronger on the higher humidity scaffolds
**Cell proliferation outcome, if any**	The highest humidity best supported cell proliferation
**Cell differentiation outcome, if any**	
**Other cell behavior data**	
**Author conclusions regarding effect of surface topography on cells**	Biological results indicate a favorable interaction between the vSMCs and the hierarchically structured fiber substrates, leading to enhanced cellular responses including cell adhesion, proliferation…
